# Short-chain fatty acid metabolites propionate and butyrate are unique epigenetic regulatory elements linking diet, metabolism and gene expression

**DOI:** 10.1038/s42255-024-01191-9

**Published:** 2025-01-09

**Authors:** Michael Nshanian, Joshua J. Gruber, Benjamin S. Geller, Faye Chleilat, Samuel M. Lancaster, Shannon M. White, Ludmila Alexandrova, Jeannie M. Camarillo, Neil L. Kelleher, Yingming Zhao, Michael P. Snyder

**Affiliations:** 1 Department of Genetics, Stanford University, School of Medicine, Stanford, CA USA; 2 Vincent Coates Foundation Mass Spectrometry Laboratory, Stanford University, Stanford, CA USA; 3 Department of Chemistry, Molecular Biosciences and Proteomics Center of Excellence, Northwestern University, Evanston, IL USA; 4 Department of Biochemistry and Molecular Genetics, Feinberg School of Medicine, Northwestern University, Evanston, IL USA; 5 Ben May Department of Cancer Research, University of Chicago, Chicago, IL USA; 6 Center for Genomics and Personalized Medicine, Stanford University School of Medicine, Stanford, CA USA

**Keywords:** Histone post-translational modifications, Epigenomics, Metabolism, Epigenetics

## Abstract

The short-chain fatty acids (SCFAs) propionate and butyrate have beneficial health effects, are produced in large amounts by microbial metabolism and have been identified as unique acyl lysine histone marks. To better understand the function of these modifications, we used chromatin immunoprecipitation followed by sequencing to map the genome-wide location of four short-chain acyl histone marks, H3K18pr, H3K18bu, H4K12pr and H4K12bu, in treated and untreated colorectal cancer (CRC) and normal cells as well as in mouse intestines in vivo. We correlate these marks with open chromatin regions and gene expression to access the function of the target regions. Our data demonstrate that propionate and butyrate bind and act as promoters of genes involved in growth, differentiation and ion transport. We propose a mechanism involving direct modification of specific genomic regions by SCFAs resulting in increased chromatin accessibility and, in the case of butyrate, opposing effects on the proliferation of normal versus CRC cells.

## Abstract (web summary)

Nshanian et al. investigate the differential effects of the short-chain fatty acids propionate and butyrate on chromatin remodelling and gain insight into the mechanisms that drive selective histone acylation.

## Main

Histone post-translational modifications (PTMs) may mediate crucial interplay between epigenetics and metabolism, with important consequences for human health and disease. In addition to canonical lysine acetylation, eight types of short-chain lysine acylations have been recently identified on histones, including propionylation (Kpr), butyrylation (Kbu), 2-hydroxyisobutyrylation, succinylation, crotonylation and β-hydroxybutyrylation^1–3^. A growing body of evidence points to a unique epigenetic regulatory role for each of these modifications^4–6^. Their presence on histones is determined by the cellular metabolic state and the availability of various forms of acyl-CoA, linking metabolism to epigenetic regulation^4,7^. The cellular concentrations of non-acetyl acyl-CoAs in turn are dependent on the presence of SCFAs. Treating cells with heavy-isotope-labelled SCFAs leads to heavy acyl labelling on histone proteins, pointing to a conversion of SCFAs to their cognate acyl-CoAs that are used as cofactors in histone acylation reactions^4,8–10^. This is accompanied by concomitant increases in the steady-state level of respective histone acylations in a dose-dependent manner^4,9,11^.

Recent studies show that major families of histone acetyltransferases (HATs) can catalyse histone acylation using acetyl-CoA, propionyl-CoA and butyryl-CoA cofactors with similar efficiencies^12–14^. For most HATs, the preference for the competing cofactor largely depends on the size of the acyl donor chain^13^. Furthermore, although almost all HATs strongly prefer acetyl-CoA, bulk levels of lysine acylations can be induced in a dose-dependent manner in response to increasing levels of given acyl-CoA metabolites^14^. Moreover, cellular concentrations of various acyl-CoAs span orders of magnitude and are closely correlated with relative abundances of acyl marks identified in vivo^14^. There is also evidence that acyl-CoAs can acylate histones non-enzymatically in vitro^14^. These findings point to a direct link between cellular metabolism and epigenetic regulation whereby differential acylation is driven by cellular concentrations of respective metabolic substrates^14–16^.

Histone acetyl marks are generally associated with active regulatory elements (effects in *cis*) that promote gene expression by neutralizing the positive charge of lysine, leading to electrostatic and structural changes in chromatin and the recruitment of readers of acylation (effects in *trans*). Chromatin immunoprecipitation followed by sequencing (ChIP-seq) experiments on Kbu, 2-hydroxyisobutyrylation, β-hydroxybutyrylation and crotonylation show an association of active regulatory elements with histone acylations and their relative levels^2,8,9,11,17^. Recent evidence demonstrates that differential acylation states correlate with distinct physiological states and biological processes involving signal-dependent gene activation, development and metabolic stress^2,8,9,11,18,19^. With respect to metabolic regulation of histone acylation, evidence points to acetyl-CoA metabolism as a key determinant of histone acetylation (Kac) in cancer cells, with changes in acetyl-CoA availability mediated by oncogenic metabolic reprogramming^20^. Under the latter process, the levels of acetyl-CoA will be reduced, leading to ketogenesis, a high NAD^+^/NADH ratio and activation of ACSS2, a known source of acyl-CoAs^11,21,22^. Acylation then occurs with acyl-CoAs other than acetyl-CoA.

Two SCFAs involved in histone acylation, propionate and butyrate, are generated in large quantities by microbial metabolism (as high as 70–100 mM in the gut lumen) and contribute to a wide array of cellular processes^23,24^. They can act as sources of energy or as substrates for histone acylation and chromatin modification by directly targeting sites or recruiting remodelling proteins^5^. Although the underlying regulatory mechanisms are largely unknown, the histone PTM state has a key role^4,5^. Importantly, the abundance of SCFAs in the microbiome from dietary fibre metabolism makes them very attractive natural therapeutic agents, especially in the context of CRC. In cancer cells, butyrate and, to a lesser extent, propionate have been shown to have antiproliferative properties that are generally attributed to the inhibition of histone deacetylation (HDAC)^25,26^. According to this model, the antiproliferative, apoptotic properties of SCFAs in cancer cells are caused by histone hyperacetylation resulting from HDAC inhibition^27^.

In this study, we sought to determine the potential regulatory function of several short-chain acyl lysine histone marks, namely propionyl and butyryl H3K18 and H4K12 in CRC versus normal cells and in vivo, as well as the effect of propionate and butyrate supplementation on chromatin accessibility and transcription. We also examined global acetylation levels as a function of propionate supplementation. We show that the unique histone marks H3K18pr, H3K18bu, H4K12pr and H4K12bu are associated with genomic regions distinct from their acetyl counterparts. On a genome-wide level, they associate with targets controlling growth, differentiation and unfolded protein response. Furthermore, they result in a more open chromatin structure and lead to the recruitment of a broad range of transcription factors. These results yield insights into how dietary factors, microbial metabolism and epigenetics are integrated to modulate tissue physiology and cancer susceptibility.

## Results

### Identification of CRC H3K18pr, H3K18bu, H4K12pr and H4K12bu marks

We focused on H3K18 and H4K12, given that acetylation at these sites has been associated with a poor outcome in CRC^28–30^. To test for Kpr and Kbu at these sites, we treated CRC cells with increasing levels (0–10 mM) of sodium propionate (NaPr) and sodium butyrate (NaBu), consistent with physiological levels of these SCFAs in fibre-supplemented colon^31,32^ (range, 1–100 mM). We then probed for the presence of these marks in acid-extracted histones using modification-specific antibodies. Immunoblots indicated the presence of Kpr and Kbu on H3K18 and H4K12 at 10 mM, and in the case of H3K18pr, at 1 mM and 10 mM propionate supplementation (Supplementary Fig. 1a,b). The results confirmed the conversion of propionate and butyrate to their cognate acyl-CoAs and their deposition as propionyl and butyryl marks on H3K18 and H4K12 in a dose-dependent manner. To further test for the presence of lysine Kpr on histones H3 and H4, we treated CRC cells with ^13^C-labelled propionate and searched for heavy peptides containing modified sites of interest by liquid chromatography–tandem mass spectrometry (LC–MS/MS).

Our results clearly indicate a direct relationship between propionate supplementation and lysine Kpr on H3 and H4, showing a steady increase in a dose-dependent manner. For example, at 10 mM supplementation, the amount of Kpr on H3K18 and H4K12 increased 1.84-fold (*P* = 0.0043) and 1.75-fold (*P* = 0.0017), respectively, compared to the control (Supplementary Fig. 1c,d). These data indicate the uptake of propionate and its deposition onto histones H3 and H4 as propionyl–lysine marks. As stated earlier, major families of HATs can catalyse histone acylation such as Kpr and Kbu with similar efficiencies^12–14^. Our results demonstrate that HAT1, known to acetylate lysine K5 and K12 sites, also mediates the incorporation of propionate into chromatin on the histone H4K12 site. Short hairpin RNA (shRNA)-induced depletion of HAT1 diminished the incorporation of propionate into the H4K12 site compared to the control shRNA (Supplementary Fig. 1e). These findings point to a direct relationship between SCFA supplementation and histone Kpr and Kbu at 1 mM and 10 mM concentrations and, more specifically, histone Kpr on H4K12 by HAT1 at 10 mM propionate supplementation.

### Acyl-CoA levels increase with SCFA supplementation

To further investigate the relationship between SCFA supplementation and the generation of the corresponding active acyl-CoA forms, we performed a quantitative metabolomics experiment using LC–MS/MS and measured the concentrations of acyl-CoA species as a function of propionate and butyrate supplementation. Our results clearly indicate a dose-dependent rise in acyl-CoA levels with increasing SCFA concentrations. For example, the amount of propionyl-CoA increased from 0.08 ng per 1 × 10^6^ cells (*P* < 0.0061) to 0.35 ng per 1 × 10^6^ cells (*P* < 0.0001) and 0.59 ng per 1 × 10^6^ cells (*P* < 0.0001) at 0.1 mM, 1 mM and 10 mM propionate supplementations, respectively, compared to the control (Supplementary Fig. 2a). Similarly, the amounts of butyryl-CoA increased from 0.04 ng per 1 × 10^6^ cells (*P* = 0.0220) to 0.08 ng per 1 × 10^6^ cells (*P* = 0.0007) at 1 mM and 10 mM butyrate supplementation, respectively (Supplementary Fig. 2b).

We then examined the levels of acetyl-CoA as a function of propionate and butyrate supplementation. With respect to propionate supplementation, our results do not show any significant increase in acetyl-CoA levels (Supplementary Fig. 2c). By contrast, butyrate supplementation led to a 2.25-fold decrease in acetyl-CoA levels at 1 mM (*P* < 0.0014) and a 3.81-fold decrease at 10 mM (*P* < 0.0002) concentrations (Supplementary Fig. 2d). Although acetyl-CoA remains the dominant species in terms of absolute amounts, supplementation with propionate and butyrate results in dose-dependent increases of their cognate acyl-CoA forms and, in the case of butyrate, nearly fourfold reduction of acetyl-CoA species. This points to a direct conversion of SCFA substrates to their corresponding active CoA forms and, in the case of butyrate (but not propionate), a shift towards reduction of acetyl-CoA at 1 mM and 10 mM concentrations in SW480 CRC cells.

The effect of butyrate as an HDAC inhibitor is well established, so we decided to investigate the effect of propionate supplementation on Kac levels in CRC cells. To that end, we examined the relative levels of acetylated versus unmodified states on several histone lysine sites as a function of propionate supplementation (0–10 mM). Our results do not show significant changes in the levels of acetylation on H3K18 or H4K12 (Supplementary Fig. 3a,b). The most abundant states on H3 and H4 were the unmodified ones, with a ratio of the unmodified to acetylated states approximately 10:1.

To test whether SCFA supplementation resembled HDAC inhibition, we compared the treatment of CRC cells with increasing levels of propionate, butyrate and trichostatin A (TSA), a potent HDAC inhibitor. TSA treatment led to a loss of cell viability at 0.1 μM and near complete cell death at 1 μM treatment (*P* < 0.0001) (Supplementary Fig. 3c). Butyrate, with its much weaker HDAC activity compared to TSA, resulted in reduced cell viability only at 10 mM supplementation (*P* = 0.0002). Propionate, on the other hand, did not affect cell viability in the 0–10 mM range but only at 100 mM (*P* < 0.0001). Both propionate and butyrate supplementations resulted in near complete loss of cell viability at 100 mM concentrations. Given the toxicity apparent at high levels, our experiments were performed with 10 mM propionate and 1 mM butyrate to avoid cytotoxicity artefacts.

### Genomic localization of H3K18pr and H4K12pr in CRC cells

**Fig. 1:**
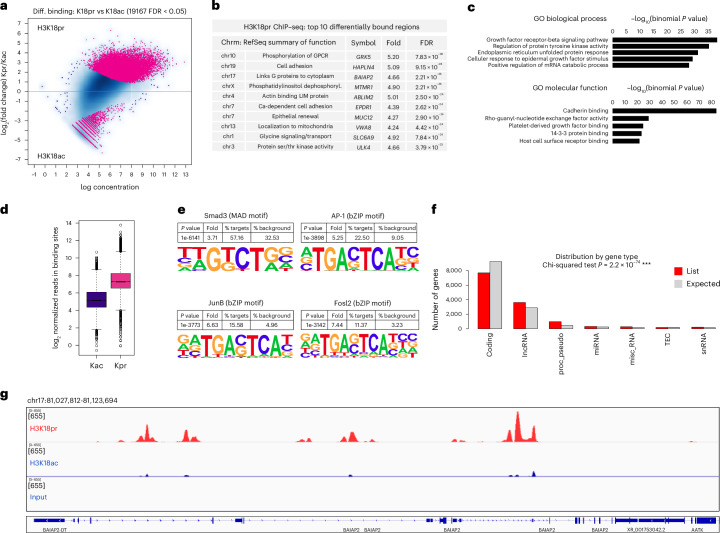
Genome-wide H3K18pr distribution. **a**, H3K18pr versus H3K18ac differential binding at 10 mM propionate treatment. Sites identified as significantly differentially bound are shown in red; *n* = 3 technical replicates for each condition. Differential binding was performed using the DiffBind package with DESeq2, using a two-sided test for both increased and decreased binding affinity between conditions followed by multiple hypothesis testing and FDR correction. **b**, Top ten differentially bound regions associated with H4K12pr, annotated to within 1 kb of the TSS, sorted by FDR-adjusted *P* value (FDR < 0.05). **c**, Top GO biological process and molecular function terms of H3K18pr-associated *cis*-regulatory elements (5 + 1 kb) determined by GREAT against a whole genome background using a binomial test over genomic regions, followed by multiple hypothesis testing using FDR-corrected *P* values (FDR < 0.05). **d**, Normalized reads in H4K12pr-associated versus H4K12ac-associated binding sites at 10 mM propionate treatment. Box plots display the minimum, first quartile (Q1, 25th percentile; bottom of the box), median (line within the box, 50th percentile), third quartile (Q3, 75th percentile; top of the box) and maximum. The whiskers extend to the most extreme data points within 1.5 × IQR (interquartile range). **e**, Differential motif analysis of H3K18pr versus H3K18ac peaks was analysed by HOMER, using a one-sided hypergeometric test for overrepresentation (enrichment) of motifs in the target sequences compared to the background, followed by multiple hypothesis testing and FDR correction. **f**, Distribution of H3K18pr peaks by gene type was measured by two-sided chi-squared test to assess whether the observed distribution significantly differs from the control distribution, without specifying a direction (enrichment or depletion), followed by multiple hypothesis testing and FDR correction. **g**, Signal tracks for regions representing *BAIAP2*. Signal intensity of peaks in 95 kb-spanning *BAIAP2* region showing H3K18pr versus H3K18ac binding at 10 mM propionate treatment with input as background. Source data

We next focused on the genome-wide distribution of H3K18pr and H4K12pr. Given the close coupling of acetylation, Kpr and Kbu, we compared differential Kpr binding to the corresponding acetyl marks. Results showed that out of 19,167 sites identified as differentially bound after propionate supplementation, 17,299 (90%) were associated with H3K18pr versus 1,868 associated with H3K18ac (false discovery rate (FDR) < 0.05) (Fig. 1a,b,g). Gene ontology (GO) analysis of Kpr-bound regions and distal *cis*-regulatory elements^33^ pointed to enrichment in epidermal growth factor stimulus and cadherin binding as well as endoplasmic reticulum unfolded protein response (Fig. 1c). KEGG pathway analysis also showed enrichment focal adhesion (110), actin cytoskeleton (115) and cancer regulatory pathways (Supplementary Fig. 4a,b). We focused on the enrichment of CRC-relevant motifs such as SMAD2/3 of the TGF-β pathway as well as AP-1, FOSL2 and JUNB. All four motifs showed enrichment in K18pr over K18ac and input, with JUNB, FOS2L and AP-1 exhibiting greater than a sixfold, sevenfold and fivefold enrichment over background, respectively (Fig. 1e). Differential binding of key Wnt/β-catenin pathway genes showed approximately threefold enrichment in *CTNNB1*, *TCF20* and *LEF1* (Supplementary Fig. 4c). We also observed a twofold to threefold enrichment in *FOS* and *JUN*. Distribution of reads over all differentially bound sites showed Kpr as having a higher mean read concentration than Kac (Fig. 1d). Hierarchical clustering of Kpr-annotated genes showed several clusters including cell projection organization and localization, while chromosomal positions and distribution by gene type showed enriched regions on chromosomes 3, 9, 17 and 19 as well as the presence of long non-coding RNAs (lncRNAs) and processed pseudogenes (Supplementary Fig. 4d,e).

**Fig. 2:**
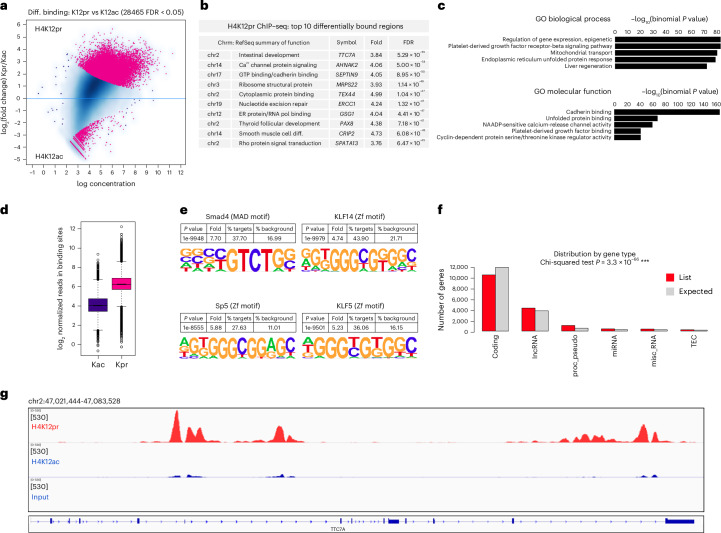
Genome-wide H4K12pr distribution. **a**, H4K12pr versus H4K12ac differential binding at 10 mM propionate treatment. Sites identified as significantly differentially bound are shown in red; *n* = 3 technical replicates for each condition. Differential binding was performed using the DiffBind package with DESeq2, using a two-sided test for both increased and decreased binding affinity between conditions, followed by multiple hypothesis testing and FDR correction. **b**, Top ten differentially bound regions associated with H4K12pr, annotated to within 1 kb of the TSS, sorted by FDR-adjusted *P* value (FDR < 0.05). **c**, Top GO biological process and molecular function terms of H4K12pr-associated *cis*-regulatory elements (5 + 1 kb) determined by GREAT against a whole genome background using a binomial test over genomic regions, followed by multiple hypothesis testing using FDR-corrected *P* values (FDR < 0.05). **d**, Normalized reads in H4K12pr-associated versus H4K12ac-associated binding sites at 10 mM propionate treatment. Box plots display the minimum, Q1, median, Q3 and maximum. The whiskers extend to the most extreme data points within 1.5 × IQR. **e**, Differential motif analysis of H4K12pr versus H4K12ac peaks was analysed by HOMER, using a one-sided hypergeometric test for overrepresentation (enrichment) of motifs in the target sequences compared to the background, followed by multiple hypothesis testing and FDR correction. **f**, Distribution of H4K12pr peaks by gene type was measured by two-sided chi-squared test to assess whether the observed distribution significantly differs from the control distribution, without specifying a direction (enrichment or depletion), followed by multiple hypothesis testing and FDR correction. **g**, Signal tracks for regions representing *TTC7A*. Signal intensity of peaks in 95 kb-spanning *TTC7A* region showing H4K12pr versus H4K12ac binding at 10 mM propionate treatment with input as background.

H4K12pr ChIP-seq identified 28,465 sites as differentially bound between Kpr and Kac, with 27,175 (95%) sites associated with Kpr (FDR < 0.05) (Fig. 2a,b,g). Annotation of genomic regions pointed to enrichment in genes controlling Rho-guanyl-nucleotide exchange activity and differentiation, cadherin and endoplasmic reticulum protein binding, as well as calcium channel signalling (Fig. 2b). As with H3K18pr, GO and KEGG analysis pointed to pathways controlling platelet-derived growth factor and cadherin binding, endoplasmic reticulum unfolded protein response and Ca^2+^ channel activity (Fig. 2c and Supplementary Fig. 5a,b). Motif analysis also showed enrichment in the SMAD family and Krüppel-like factors KLF5 and KLF14, which are known for their regulatory role as recruiters of other transcription factors (Fig. 2e). Hierarchical clustering showed several clusters such as cellular catabolic processes, protein transport and cellular localization, and chromosomal positions and distribution by gene type showed enrichment along chromosomes 7, 9, 11 and 17 as well as the presence of lncRNAs and processed pseudogenes (Supplementary Fig. 5d,e and Fig. 2f). H4K12pr peak distributions, regulated genes and GO enrichment analysis showed generally similar results with H3K18pr analyses, including prioritization of Wnt/β-catenin, TGF-β and FOS, JUN and MYC transcription factors (Fig. 2a,c,e and Supplementary Fig. 5c).

We then integrated our Kpr ChIP-seq results with assay for transposase-accessible chromatin followed by sequencing (ATAC-seq) and RNA sequencing (RNA-seq) results in CRC cells to determine intersecting genomic coordinates and overlapping annotated genes. Genomic coordinates present in both Kpr ChIP-seq and propionyl ATAC-seq datasets (*n* = 4,391*, P* = 4.75 × 10^−4^ and *n* = 4,038, *P* = 2.31 × 10^−38^, respectively) showed enrichment in β-catenin–TCF complex assembly, negative regulation of MAPK cascade and actin filament organization (Supplementary Fig. 6a,b,e,f). There was also enrichment in Rho-guanyl-nucleotide exchange factor activity and proteins involved in cell adhesion. Genes relevant to the identified pathways and to CRC in particular also showed increased accessibility by ATAC-seq, indicating more open chromatin structure following propionate supplementation (Supplementary Fig. 6c,d,g,h). Integration with propionyl RNA-seq showed overlap between both H3K18pr and H4K12pr targets and upregulation (*n* = 1,528, *P* = 7.10 × 10^−154^ and *n* = 1,585, *P* = 1.69 × 10^−147^) as well as downregulation (*n* = 1,015, *P* = 3.29 × 10^–12^ and *n* = 1,317, *P* = 1.35 × 10^–167^) of gene expression (Supplementary Fig. 7a,c,e,g). GO analysis of Kpr targets and upregulated genes identified pathways in differentiation, localization as well as actin filament-based processing, while downregulated genes were involved in the mitotic cell cycle, RNA metabolism and processing (Supplementary Fig. 7b,d,f,h). This points to a mechanism through which propionate affects the CRC epigenetic regulatory landscape to promote growth, differentiation and localization over regulation of cell cycle and RNA processing.

### Genomic localization of H3K18bu and H4K12bu in CRC cells

We next examined Kbu marks on the same sites to gain functional insight and observe butyrate supplementation (1 mM) effects on chromatin structure and accessibility. Butyrate has been shown to activate the TGF-β pathway^34^ and the Wnt signalling pathway genes in CRC in particular^35^. ChIP-seq experiments on Kbu on H4K5 and H4K8 in the context of sperm cell differentiation show histone Kbu as a direct stimulator of transcription while also competing with acetylation in chromatin reorganization^4,17^. H3K14bu ChIP-seq in mouse livers was recently shown to be associated with transcriptionally active chromatin and specifically with carboxylic acid and lipid metabolism^36^.

Compared to Kpr, fewer differentially bound regions were associated with H3K18bu and H4K12bu: 2,305 and 793, respectively (Supplementary Figs. 8a and 9a). Interestingly, top-scoring sites associated with both Kbu marks were lncRNAs *PRNCR1* and *PCAT1*, enhancers in the same chromosomal region as *MYC*, shown to have a pivotal oncogenic role in CRC^37,38^. *PRNCR1* showed a 3.4-fold (FDR = 2.25 × 10^−9^) and 2.9-fold (FDR = 3.14 × 10^−77^) increase in H3K18bu and H4K12bu binding, respectively (Supplementary Figs. 8b,f and 9b), whereas *PCAT1* showed a 4.19-fold increase in H3K18bu (FDR = 6.79 × 10^−16^) and a 2.8-fold increase in H4K12bu binding (FDR = 1.57 × 10^−12^). GO enrichment, differential motif analysis and integration with butyryl ATAC-seq results identified pathways similar to Kpr and propionyl ATAC-seq (Supplementary Figs. 8c–e, 9c–f and 10d–g).

**Fig. 3:**
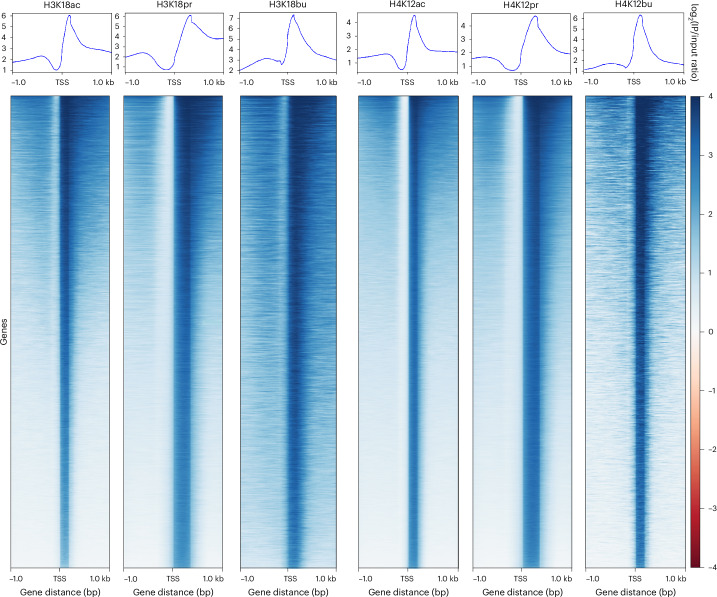
TSS distribution profiles of H3K18ac/pr/bu-associated and H4K12ac/pr/bu-associated ChIP-seq peaks as a function of read coverage. Upper panels, aggregate read density profile plots of genomic region distributions within ±1 kb of the TSS as a function of log_2_(IP/input ratio). Lower panels, read density heatmaps of gene distributions with maximum (*z* = 4) and minimum (*z* = −4) values of heatmap intensities. Plots generated by deepTools.

**Extended Data Fig. 1:**
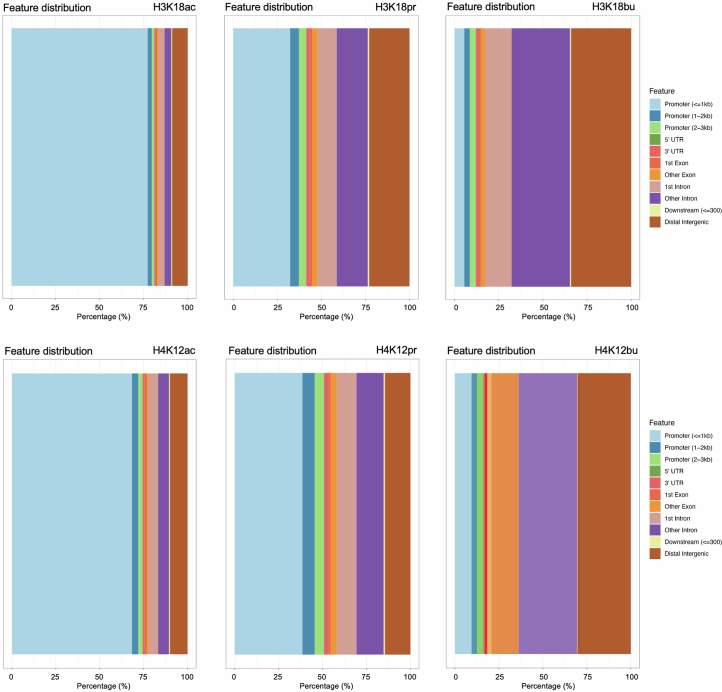
Feature distribution of H3K18ac/pr/bu (top panel) and H4K12ac/pr/bu (bottom panel) associated regions (+/- 3 Kb of TSS). H3K18ac/H4K12ac annotations were taken from ChIP-seq data that was generated without any treatment. H3K18pr/H4K12pr and H3K18bu/H4K12bu ChIP-seq experiments were performed following 10 mM NaPr, and 1 mM NaBu treatments, respectively. The x-axis provides the percentage of sites while colored regions represent distance from TSS. Results obtained using ChIPSeeker R package. Source data

Next, we visualized the read coverage over genomic regions 1 kB upstream and downstream of the transcription start site (TSS) for all our acyl lysine histone marks to evaluate global enrichment across all TSSs (Fig. 3). In each case, we observed considerable enrichment of our marks proximal to the TSS and beyond, with increases in read density over input proportional to the length of the acyl lysine chain. Propionyl and butyryl marks showed significantly higher density distributions in the positive, downstream of TSS direction compared to their acetyl counterparts. All three types of acyl marks showed consistency in the distribution profiles (Fig. 3, upper panels) and heatmap densities (Fig. 3, bottom panels). These results indicate that Kpr and Kbu marks increased chromatin accessibility compared to Kac. Feature distribution of differentially bound genes (±3 kB of TSS) also showed increased chromatin accessibility and consistency in feature distributions among the three types of acyl marks, with a shift towards distal intergenic regions in Kpr and Kbu (Extended Data Fig. 1). These results indicate a greater role of *cis*-regulatory elements and a more open chromatin structure.

**Extended Data Fig. 2:**
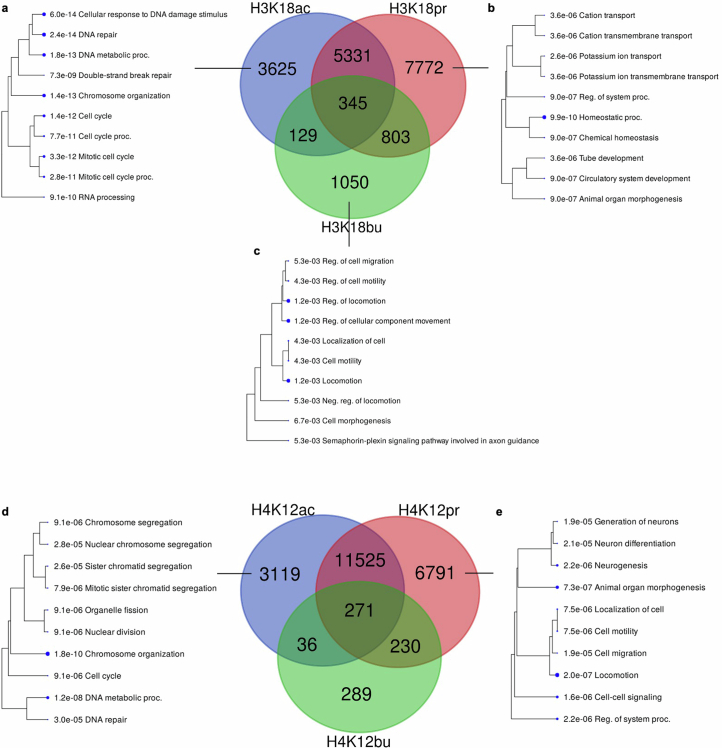
Kac/pr/bu annotated features overlap. **a, d** Hierarchical clustering of GO ‘Biological Process’ terms associated with Kac but not Kpr or Kbu. **b, e** Hierarchical clustering of GO ‘Biological Process’ terms associated with Kpr but not Kac or Kbu. **c** Hierarchical clustering of GO ‘Biological Process’ terms associated with Kbu but not Kac or Kpr. Hierarchical clustering of the pathways was performed using ShinyGO. Pathways were clustered together based on shared genes and gene enrichment analysis was performed using two-sided Fisher's exact test, and FDR correction was applied to adjust for multiple comparisons in the pathway analysis and hierarchical clustering. Size of dots indicates statistically significant FDR adjusted (FDR < 0.05) *P* values. Source data

**Extended Data Fig. 3:**
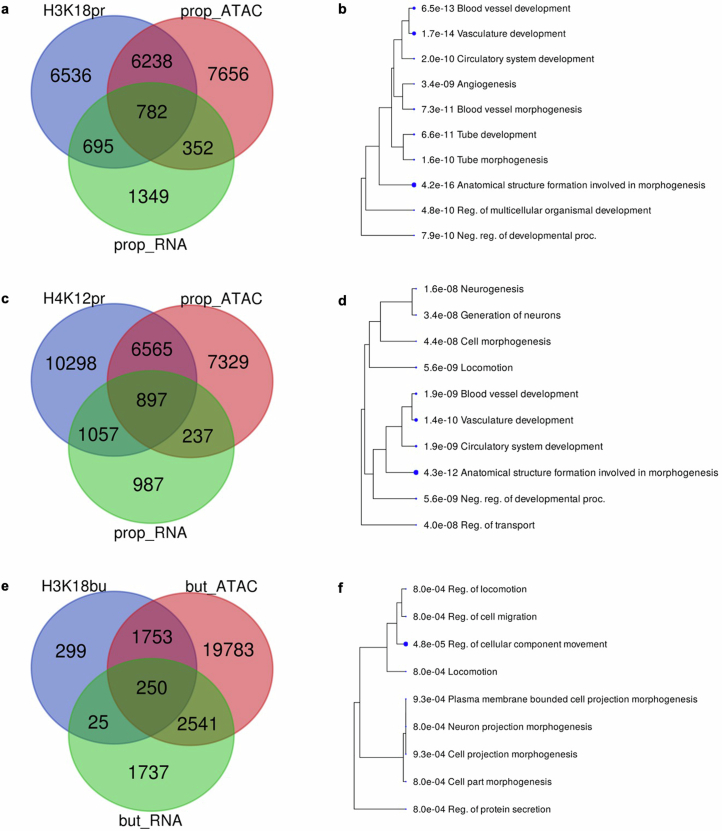
Kpr/bu and ATAC/RNA-seq shared annotated features. **a** H3K18pr, propionyl ATAC/RNA-seq shared annotated features. **b** Hierarchical cluttering of GO ‘Biological Process’ terms associated 782 shared features. **c** H4K12pr, propionyl ATAC/RNA-seq shared features. **d** Hierarchical clustering of GO ‘Biological Process’ terms associated with 897 shared features. Hierarchical clustering of the pathways was performed using ShinyGO. Pathways were clustered together based on shared genes and gene enrichment analysis was performed using two-sided Fisher's exact test, and FDR correction was applied to adjust for multiple comparisons in the pathway analysis and hierarchical clustering. Size of dots indicates statistically significant FDR adjusted (FDR < 0.05) *P* values. **e** H3K18bu butyryl ATAC/RNA-seq shared features. **f** Hierarchical cluttering of GO ‘Biological Process’ terms associated with 250 shared features. Source data

Last, we examined functional differences between the three types of acyl marks by examining annotated genes that were unique to each (Extended Data Fig. 2). Terms unique to Kac marks pointed to regulation of cell cycle and chromosome organization (Extended Data Fig. 2a,d). By contrast, terms unique to Kpr pointed to regulation of cation transport, organ morphogenesis and locomotion (Extended Data Fig. 2b,e). Elements unique to Kbu were associated almost exclusively with regulation of cell motility, migration and locomotion (Extended Data Fig. 2c). The results indicate that although there are some functional similarities between Kpr and Kbu marks, they are distinct from Kac. Comparison of shared annotated features between Kpr and Kbu marks and ATAC-seq and RNA-seq results revealed many of the same pathways, highlighting their functional similarities as well as their differences (Extended Data Fig. 3).

### SCFA increase chromatin accessibility in CRC cells

To address the effect of propionate and butyrate on chromatin accessibility at a more global level, we performed differential ATAC-seq. We reasoned that propionate and butyrate supplementation would result in greater chromatin accessibility. Out of a total of 22,238 regions identified as differentially accessible, 18,404 (83%) sites showed positive fold change in the propionate-treated group compared to 3,834 in the untreated group (FDR < 0.05) (Supplementary Fig. 10a, top). Differentially accessible genes and GO pathways associated with propionate treatment were consistent with Kpr ChIP-seq results, particularly with respect to genes controlling cell–substrate adhesion (*COL26A1*, 2.82-fold, FDR = 8.78 × 10^−37^) as well as epithelial development (*KLF2*, 3.39-fold, FDR = 6.09 × 10^−37^) and β-catenin–TCF complex assembly (Supplementary Fig. 10b, top). Sites that showed a reduction in accessibility were involved in cell cycle G1 arrest (*CDKN1A*, −1.78-fold, FDR = 9.04 × 10^−14^) as well regulation of cell cycle and cell proliferation (*ZNF703*, −1.74-fold, FDR = 1.41 × 10^−11^) (Supplementary Fig. 10a, top). Among differentially bound sites overall, the propionate-treated group had a higher mean read concentration, indicating increased binding affinity under more open chromatin (Supplementary Fig. 10c, top).

By contrast, over three times as many sites were identified with butyryl ATAC–seq compared to propionyl ATAC-seq. Out of 71,318 sites identified as differentially accessible, 38,089 (53%) showed positive fold change in the treated group and 33,229 underwent negative fold change in the untreated group (Supplementary Fig. 10a, bottom). Genomic annotations identified genes involved in muscle contraction (*SSPN*, 2.31-fold, FDR = 5.41 × 10^−136^), axon guidance and differentiation (*SEMA3D*, 2.38-fold, FDR = 5.16 × 10^−131^) as well as actin and microtubule binding (*MYO3B*, 2.41-fold, FDR = 8.11 × 10^−99^) (Supplementary Fig. 10b, bottom). GO pathway analysis showed enrichment in mesenchymal cell proliferation, metanephros development and fibroblast apoptotic process, as seen with Kbu ChIP-seq (Supplementary Fig 10b, bottom and Supplementary Figs. 8c and 9c).

Among sites that lost accessibility were genes encoding a zinc finger transcription factor required for DNA damage-induced p53 activation (*CXXC5*, −2.06-fold, FDR = 1.26 × 10^−187^) and a G protein subfamily member mediating transmembrane signalling (*GNAZ*, −3.04-fold, FDR = 4.76 × 10^−160^). Among differentially accessible sites overall, the butyrate-treated group did not have a significantly higher mean read concentration, indicating that the increased accessibility in the treated group was offset by decreased accessibility in the untreated group (Supplementary Fig. 10c, bottom).

### SCFA affect gene expression in CRC cells

**Fig. 4:**
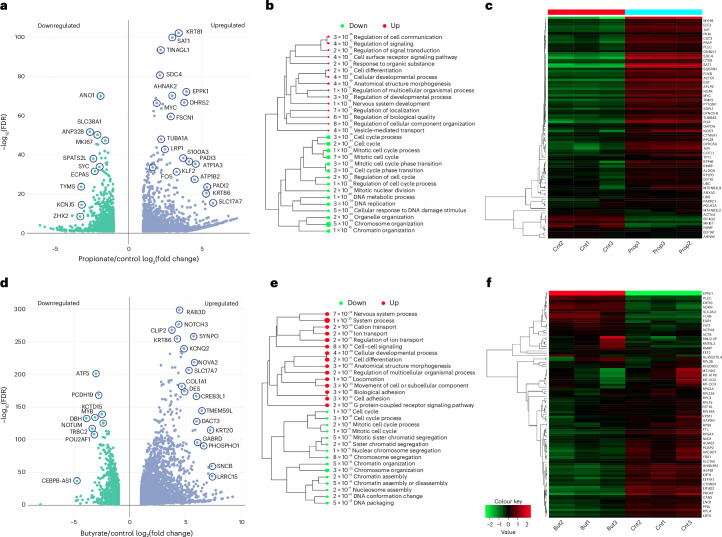
Propionyl and butyryl differential gene expression by RNA-seq. **a**, Volcano plot showing gene upregulation versus downregulation in 10 mM propionate-treated versus control groups; *n* = 3 technical replicates for each condition. Differential expression analysis performed by DESeq2 **b**, Hierarchical clustering of GO biological process terms of upregulated versus downregulated pathways in propionate-treated versus control groups. Hierarchical clustering of the pathways was performed using ShinyGO. Pathways were clustered together based on shared genes. Gene enrichment analysis was performed using a two-sided Fisher’s exact test, and FDR correction was applied to adjust for multiple comparisons in the pathway analysis and hierarchical clustering. Size of dots indicates statistically significant FDR-adjusted (FDR < 0.05) *P* values. **c**, Heatmaps of the 50 most variable genes in propionate-treated versus control (Cnt) groups. **d**, Volcano plot showing gene upregulation versus downregulation in 1 mM butyrate-treated versus control groups. **e**, Hierarchical clustering of GO biological process terms of upregulated versus downregulated pathways in butyrate-treated versus control groups. **f**, Heatmaps of the 50 most variable genes in butyrate-treated versus control groups.

To gain a more complete picture of SCFA-induced alterations on the regulatory landscape of CRC, we examined global changes in gene expression by bulk RNA-seq. Differential gene expression analysis following propionate supplementation identified 2,027 upregulated and 1,151 downregulated genes (Fig. 4a). Among the genes that showed significant upregulation and were also identified as Kpr targets and exhibited increased accessibility by ATAC-seq were *SAT1* (2.99-fold FDR = 2.04 × 10^−100^), *AHNAK2* (2.98-fold, FDR = 8.71 × 10^−71^), *DHRS2* (3.80-fold, FDR = 2.44 × 10^−67^), *KLF2* (3.28-fold, FDR = 4.95 × 10^−32^) as well as *MYC* and *FOS* (Supplementary Figs. 4c and 5c). Among genes that underwent downregulation were mainly those involved in cell proliferation (*ANP32B*, −2.63-fold, FDR = 2.01 × 10^−52^) and cell cycle progression (*MKI67*, −1.57-fold, FDR = 5.90 × 10^−48^). Hierarchical clustering showed upregulation in receptor signalling, development and anatomical structure morphogenesis and downregulation in cell cycle, cell division and chromatin organization (Fig. 4b). The heatmap of the top 50 most variable genes showed enrichment in *MYC, JUN* and *AHNAK2* (Fig. 4c).

Similarly, butyrate supplementation led to upregulation of genes controlling ion transport, anatomical structure morphogenesis and cell adhesion and downregulation in cell cycle and chromatin assembly (Fig. 4d–f). Taken together, our data point to a mechanism whereby the antiproliferative properties of propionate and butyrate in CRC can be attributed to their dysregulation of key CRC oncogenes such as *MYC*, *FOS* and *JUN* as well their simultaneous triggering of downregulation of genes controlling cell cycle and cell division.

**Fig. 5:**
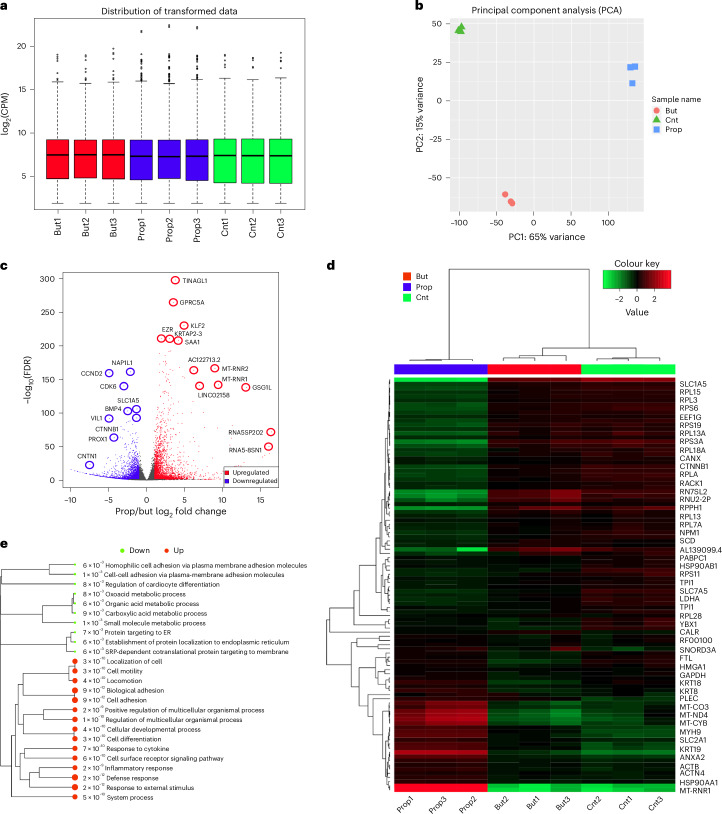
Propionyl/butyryl differential RNA-seq. **a**, Distribution of log_2_(CPM)-transformed expression data for all conditions; *n* = 3 technical replicates for each condition. Normalization of raw counts performed by ‘cpm’ analysis in edgeR. CPM, counts per million. Box plots display the minimum, Q1, median, Q3 and maximum. The whiskers extend to the most extreme data points within 1.5 × IQR. **b**, Principal component analysis of log_2_(CPM)-transformed expression data for all conditions. **c**, Volcano plot of propionyl versus butyryl differential expression. **d**, Heatmaps of the 50 most variable genes for all three conditions. **e**, Hierarchical clustering of GO biological process terms of differentially expressed pathways in propionyl versus butyryl RNA-seq. Hierarchical clustering of the pathways was performed using ShinyGO. Pathways were clustered together based on shared genes, and gene enrichment analysis was performed using a two-sided Fisher’s exact test. FDR correction was applied to adjust for multiple comparisons in the pathway analysis and hierarchical clustering. Size of dots indicates statistically significant FDR-adjusted (FDR < 0.05) *P* values.

To examine the differences in expression that were unique to propionate and butyrate, we compared differential gene expression under both types of enrichments. Density distributions of counts were consistent across conditions and replicates within each condition (Fig. 5a). Principal component analysis showed close clustering of replicates within each condition, with the most variation seen between different conditions forming separate clusters (Fig. 5b). Differential expression following propionate versus butyrate supplementation showed 3,082 upregulated and 2,783 downregulated genes (Fig. 5c). The heatmap of the top 50 most variable genes and hierarchical clustering of GO biological process terms showed preferential enrichment of organic and carboxylic acid metabolism associated with butyrate supplementation, whereas propionate supplementation showed enrichment in cell motility and locomotion as well as cellular development and differentiation (Fig. 5d,e).

**Extended Data Fig. 4:**
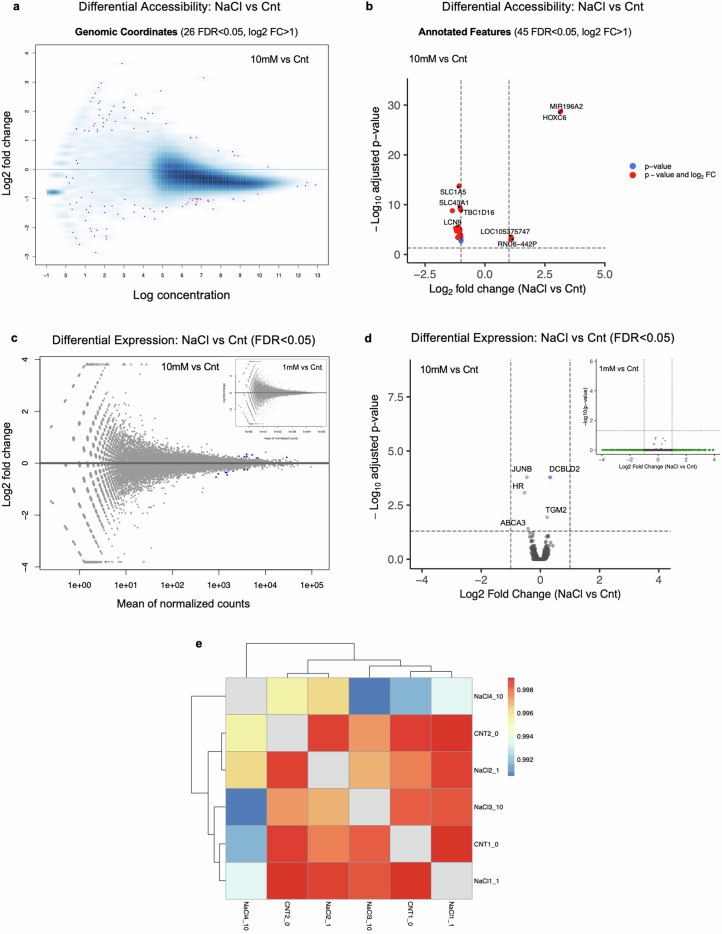
Effect of Na^+^ on differential accessibility and expression. **a** ATAC-seq MA plot of differential accessibility in 10 mM NaCl-treated vs untreated cells (SW480). Differential analysis was performed by DESeq2 using two-sided Wald tests to identify differentially expressed or accessible genes. The results were then adjusted for multiple comparisons using the FDR correction method. Sites identified as significantly differentially accessible (FDR<0.05, log2 FC>1) are shown in red. n = 3 experimental replicates for each condition. **b** Volcano plot of differential accessibility in 10 mM NaCl-treated vs untreated cells. **c** RNA-seq MA plot of differential expression in 10 mM NaCl treated vs untreated cells. Sites identified as significantly differentially expressed (FDR<0.05) are shown blue (*Insert*: 1 mM vs Cnt). **d** Volcano plot of differential expression in 10 mM NaCl-treated vs untreated cells. Sites identified as significantly differentially expressed by FDR only (FDR<0.05) are shown blue (*Insert*: 1 mM vs Cnt). **e** Correlation heatmap of RNA-seq data showing clustering replicates from 1 and 10 mM NaCl-treated vs untreated groups. Normalized measurement of the covariance between replicates is expressed by Pearson’s correlation coefficient. Source data

To address the osmolarity of the sodium counterion and potential off-target effects of sodium resulting from NaPr and NaBu supplementation at 1 mM and 10 mM concentrations used in our differential chromatin accessibility by ATAC-seq and differential expression by RNA-seq data, we performed those experiments in the presence and absence of 1 mM and 10 mM sodium chloride (NaCl) rather than NaPr and NaBu. Differential accessibility by ATAC-seq at 1 mM NaCl supplementation versus control did not generate sufficient contrast between the two conditions for differential expression analysis. At 10 mM NaCl versus control, 26 sites were identified (FDR < 0.05, log_2_(fold change) > 1) as differentially accessible with only four regions showing positive fold change in accessibility as a result of 10 mM NaCl supplementation (Extended Data Fig. 4a). Three of those regions showed only very modest fold change (log_2_(fold change) = 1.08–1.11, FDR = 0.0012–00029) with only one region showing 3.15-fold change in accessibility (FDR = 2.06 × 10^−29^).

Annotation of differentially bound regions at 10 mM NaCl supplementation identified region 12q13.13, mapping to *HOX6*, a member of a homeobox family of transcription factors, and an adjacent ncRNA gene, *MIR196A2I*, affiliated with the miRNA class (3.15-fold, FDR = 2.06 × 10^−29^) (Extended Data Fig. 4b). The regions that underwent negative fold change (or were preferentially enriched in the control group) upon 10 mM NaCl supplementation above the significance threshold were *SLC1A5* (−1.08-fold, FDR = 1.81 × 10^−14^) and *SLC43A* (−1.04-fold, FDR = 4.13 × 10^−10^), genes encoding sodium-coupled neutral amino acid transporters; *TBC1D16* (−1.01-fold, FDR = 1.20 × 10^−9^), a TBC1 domain family member involved in GTPase activator activity and regulation of receptor recycling; and *LCN9* (−1.1-fold, FDR = 3.44 × 10^−6^), a member of the lipocalin family involved in binding and transport of small hydrophobic ligands. Overall, at 10 mM NaCl supplementation, the number of differentially accessible regions by ATAC-seq that showed positive fold change or enrichment over the control above the significance threshold was insufficient to account for any potential off-target effects caused by sodium in NaPr and NaBu supplementation.

Similarly, differential expression analysis by RNA-seq in the presence and absence of 1 mM and 10 mM NaCl did not result in any significant contrast between NaCl and control conditions (Extended Data Fig. 4c,d). No genes were identified above the significance threshold at 1 mM versus control (Extended Data Fig. 4c,d, insert) and only two were identified that showed slight preferential enrichment in the 10 mM NaCl condition: *DCBLD2* (0.31-fold, FDR = 0.00016), a gene involved in negative regulation of cell growth and wound healing, and *TGM2* (0.22-fold, FDR = 0.01147), a gene involved in GTP binding and protein–glutamine gamma-glutamyltransferase activity. Genes showing negative fold change in expression were *JUNB* (−0.45-fold, FDR = 0.00016) and *HR* (−0.54-fold, FDR = 0.000838), genes involved in DNA-binding transcription factor activity and transcription corepressor activity.

Given that all immunoprecipitation-based experiments were carried out under the same NaPr and NaBu enrichment conditions as the controls and only acyl lysine antibodies were compared, the effects of the sodium counterion were thus normalized across all samples and conditions in our Kpr and Kbu ChIP-seq and CUT&Tag experiments. Therefore, only ATAC-seq and RNA-seq experiments that were carried out at different treatment conditions were further investigated for potential off-target effects owing to the sodium counterion. Based on the significance threshold (FDR < 0.05, log_2_(fold change) > 1) for differential accessibility and expression, we did not see evidence of off-target effects caused by sodium that would affect the results in our ATAC-seq and RNA-seq experiments. Furthermore, the RNA-seq correlation heatmap of 1 mM and 10 mM NaCl groups versus the control showed clustering of replicates under different conditions (Extended Data Fig. 4e). Thus, there is no significant statistical difference in expression between the 1 mM and 10 mM NaCl supplemented groups and the control in SW480 cells.

### Genomic localization of Kbu marks in normal versus cancer cells

**Extended Data Fig. 5:**
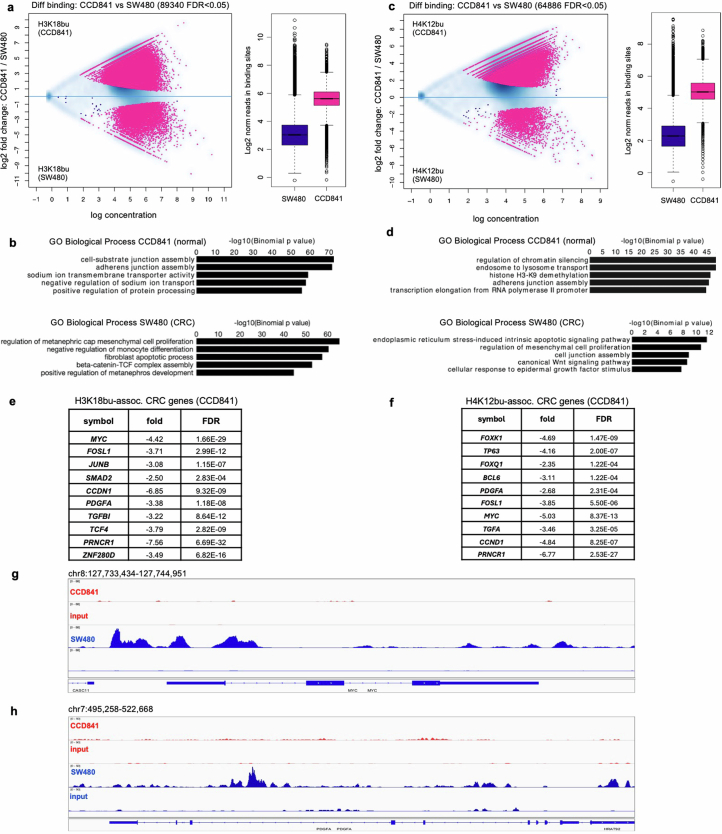
H3K18bu vs ac and H4K12bu vs ac differential binding from mouse intestine on HSF + 5 % arabinoxylan diet. **a** H3K18bu differential binding in cancer (SW480) vs normal (CCD841) cells and normalized reads in H3K18bu-associated binding sites following 1 mM NaBu treatment. Sites identified as significantly differentially bound are shown in red. n = 3 technical replicates for each condition. Differential binding was performed by DiffBind package with DESeq2 using a two-sided tests for both increased and decreased binding affinity between conditions followed by multiple hypothesis testing and FDR correction (FDR < 0.05). Box plots display: The minimum, first quartile (Q1, 25^th^ percentile), median, third quartile (Q3, 75^th^ percentile), and maximum. The bottom of the box is Q1 and the top of the box is Q3. The line within the box represents the median (50^th^ percentile) value. The whiskers extend to the most extreme data points within 1.5 times the IQR (interquartile range). **b** Top GO Biological Process terms for H3K18bu-associated *cis*-regulatory elements in cancer vs normal cells determined by GREAT against a whole genome background using a binomial test over genomic regions, followed by multiple hypothesis testing using FDR corrected *P* values (FDR < 0.05). **c** H4K12bu differential binding in cancer vs normal cells and normalized reads in H4K12bu associated binding sites following 1 mM NaBu treatment. **d** Top GO Biological Process terms for H4K12bu-associated *cis*-regulatory elements in cancer vs normal cells. **e, f** CRC differentially bound genes associated with H3K18bu/H4K12bu. **g, h** Signal tracks showing differential binding in *MYC* and *PDGFA* regions in cancer vs normal cells. Source data

We next examined the differences in the genome-wide distribution of Kbu marks in CRC (SW480) versus normal (CCD841) cells following 1 mM butyrate supplementation, particularly in relation to CRC-relevant genes. Out of 89,340 targets associated with H3K18bu differential binding in the two cell lines, 81,999 (92%) sites had higher binding affinity in normal cells compared with 7,341 sites in cancer cells (FDR < 0.05) (Extended Data Fig. 5a). GO biological process analysis in the normal cell line showed enrichment in cell–substrate and adherens junction assembly, as well as ion transport and protein processing (Extended Data Fig. 5b, top). By contrast, the same mark in the cancer cell line showed enrichment in mesenchymal cell proliferation, β-catenin–TCF complex assembly and fibroblast apoptotic process (Extended Data Fig. 5b, bottom). Similarly, out of 64,886 targets associated with H4K12bu, 61,978 (96%) sites had higher binding affinity in normal cells compared to 2,908 sites in cancer cells (Extended Data Fig. 5c). GO biological process analysis in normal cells also showed enrichment in adherens junction assembly and transport but also regulation of chromatin silencing and H3K9 demethylation (Extended Data Fig. 5d, top). By contrast, GO analysis in the cancer cell line showed enrichment in mesenchymal cell proliferation, Wnt signalling pathway and endoplasmic reticulum stress-induced apoptotic signalling (Extended Data Fig. 5d, bottom).

Although both marks were associated with adherens and cell junction assembly, in SW480 cancer cells they were associated with mesenchymal cell proliferation, Wnt/β-catenin signalling and apoptotic processes, whereas in normal cells they showed association with transmembrane ion and endosome to lysosome transport along with protein processing. Moreover, following butyrate supplementation, many of the CRC-relevant genes monitored throughout the study, such as *MYC* and *FOSL1*, showed a threefold to sevenfold reduction in Kbu binding affinity in the normal cell line compared to the CRC cell line (Extended Data Fig. 5e–h).

### Genomic localization of Kbu marks in mouse intestines

**Fig. 6:**
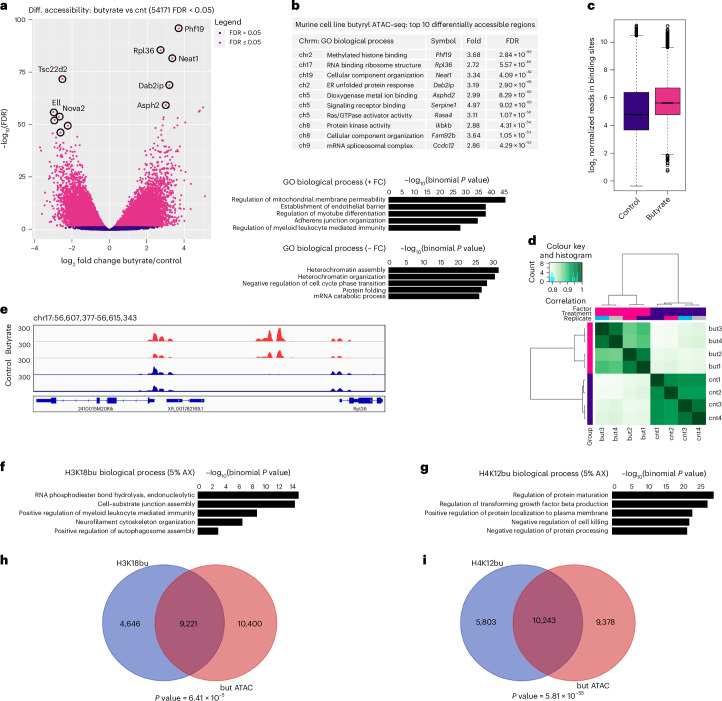
Murine cell line butyryl ATAC-seq and Kbu associated targets in mouse intestines. **a**, Differential accessibility at 1 mM butyrate supplementation. Sites identified as significantly differentially accessible are shown in red; *n* = 4 technical replicates for each condition. Differential accessibility was performed using the DiffBind package with DESeq2, using a two-sided test for both increased and decreased binding affinity between conditions followed by multiple hypothesis testing and FDR correction. **b**, Top ten differentially accessible regions associated with butyrate supplementation sorted by FDR-adjusted *P* value (FDR < 0.05) and top GO biological process terms associated with positive versus negative fold change determined by GREAT against a whole genome background using a binomial test over genomic regions, followed by multiple hypothesis testing with FDR-corrected *P* values (FDR < 0.05). **c**, Normalized reads in binding sites at butyrate supplementation. Box plots display the minimum, Q1, median, Q3 and maximum. The whiskers extend to the most extreme data points within 1.5 × IQR. **d**, Correlation heatmap showing clustering of replicates from butyrate-supplemented versus untreated group. **e**, Signal tracks representing differential accessibility between butyrate-supplemented and untreated groups. **f**, Top GO biological process terms associated with H3K18bu in mouse intestines (5% arabinoxylan (AX)). **g**, Top GO biological process terms associated with H4K12bu in mouse intestines (5% AX). **h**, Annotated peak overlap between mouse intestinal H3K18bu (5% AX) targets and butyryl ATAC-seq in murine cells. **i**, Annotated peak overlap between mouse intestinal H4K12bu (5% AX) targets and butyryl ATAC-seq in murine cells.

**Extended Data Fig. 6:**
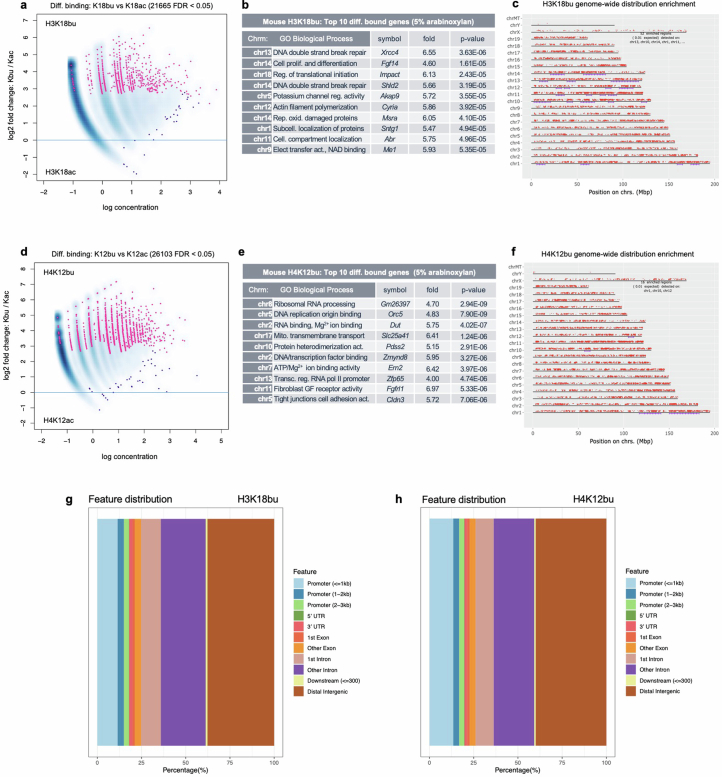
H3K18bu vs ac and H4K12bu vs ac differential binding from mouse intestine on HSF + 5 % arabinoxylan diet. **a, b, d, e** Sites identified as significantly differentially bound are shown in red. n = 3 biological replicates for each condition. Differential binding was performed by DiffBind package with DESeq2 using a two-sided tests for both increased and decreased binding affinity between conditions followed by multiple hypothesis testing and FDR correction. **c, f** Chromosomal positions of H3K18bu and H4K12bu-associated regions represented by red dots. Purple lines represent statistically significant enrichment compared to input. The genome was scanned with a sliding window (size 6 Mb) further subdivided into 2 equal-sized steps for sliding. Within each window a hypergeometric test was used to test for enrichment over all protein-coding genes in the genome. FDR adjusted *P* value cutoff for window was 1E-05. Gene chromosomal mapping performed using ShinyGO. **g, h** Feature distribution of H3K18bu and H4K12bu bound regions (+/− 3 Kb of TSS). The x-axis provides the percentage of sites while colored regions represent distance from TSS. Results obtained using ChIPSeeker R package. Source data

To further investigate the link between dietary fibre metabolism, chromatin accessibility and histone Kbu, we performed ATAC-seq in CT26 mouse colorectal cells and cleavage under targets and tagmentation (CUT&Tag) on large intestine tissues from mice fed chow containing the dietary fibre arabinoxylan (5% w/w) (see Methods). First, we examined differential chromatin accessibility following 1 mM butyrate supplementation in CT26 murine CRC cells. Out of 54,171 sites identified as differentially accessible, 39,956 (74%) gained accessibility following butyrate supplementation compared to 14,215 sites in the untreated group (FDR < 0.05) (Fig. 6a). Peak distributions, regulated genes and GO enrichment analysis showed results similar to those in SW480 and CCD841 cell lines (Fig. 6a–e). Differential Kbu binding analysis of mouse intestines on a 5% arabinoxylan diet by CUT&Tag identified 21,665 sites associated with H3K18bu versus Kac and 26,103 sites associated with H4K12bu versus Kac (FDR < 0.05) (Extended Data Fig. 6a,b,d,e). GO analysis and feature distributions were in agreement with Kbu ChIP-seq results in SW480 cells (Extended Data Fig. 6c,f,g,h). Annotation of top H3K18bu-associated regions identified genes involved in cell proliferation and differentiation (*Fgf14*, 4.60-fold, *P* = 1.61 × 10^−5^) as well as ion channel regulatory activity (*Akap9*, 5.72-fold, *P* = 3.55 × 10^−5^) and actin filament polymerization (*Cyria*, 5.86-fold, *P* = 3.92 × 10^−5^) (Extended Data Fig. 6b). Similarly, top H4K12bu-associated regions were involved in fibroblast growth factor receptor activity (*Fgfrl1*, 6.97-fold, *P* = 5.33 × 10^−6^) as well as tight junction cell adhesion activity (*Cldn3*, 5.72-fold, *P* = 5.33 × 10^−6^) (Extended Data Fig. 6e). H3K18bu GO analysis identified pathways controlling cell–substrate junction assembly, cytoskeletal organization as well as autophagosome assembly (Fig. 6g). H4K12bu GO analysis identified pathways controlling protein processing, membrane localization and TGF-β production (Fig. 6h). Comparing mouse butyryl ATAC-seq in cells following butyrate supplementation to H3K18bu targets in vivo revealed 9,221 (72%) overlapping elements out of 13,867 (*P* = 6.41 × 10^−5^) (Fig. 6i). Comparing mouse butyryl ATAC-seq results following butyrate supplementation to H4K12bu targets in vivo revealed 10,243 (64%) overlapping elements out of 16,046 (*P* = 5.81 × 10^−55^) (Fig. 6j).

## Discussion

**Extended Data Fig. 7:**
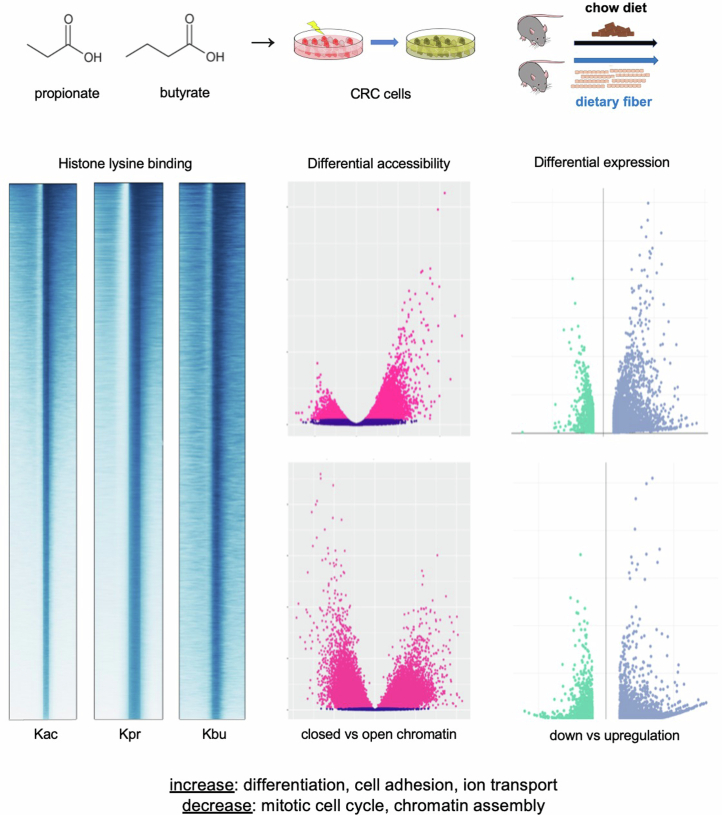
Overview of SCFAs propionate and butyrate as regulatory elements affecting histone binding, chromatin accessibility and gene expression. Upper panels: *In vitro* cellular perturbations and *in vivo* fiber supplementation experimental designs. Bottom panels: Changes in TSS distribution profiles, differential chromatin accessibility and expression following SCFA supplementation. Top and Bottom volcano plots showing differential accessibility and expression following propionate and butyrate supplementation, respectively. NIAID Visual & Medical Arts. 09/26/2024. Grey laboratory mouse. NIAID BIOART Source. bioart.niaid.nih.gov/bioart/280. Source data

The SCFAs propionate and butyrate are produced by the microbiome and have broad biological effects. To gain insights into how they may directly affect gene regulation and expression, we combined histone PTM profiling by LC–MS/MS with ChIP-seq, CUT&Tag, ATAC-seq and RNA-seq to understand the epigenetic regulatory function of these SCFA metabolites in CRC versus normal cells and in vivo (Extended Data Fig. 7). We identified the potential functional role of several propionyl–lysine and butyryl–lysine modifications on H3 and H4 by identifying their genomic regions of interaction as well as their biological pathways. We have also shown the effect of these marks in promoting chromatin accessibility and changes in gene expression.

Our results link SCFA supplementation to epigenetic regulation of gene expression by non-canonical histone modifications and increased chromatin accessibility. In the context of CRC, SCFA supplementation led to homeostatic dysregulation by hyperactivation of Wnt/β-catenin as well as TGF-β signalling pathways and activation of *MYC, FOS* and *JUN* oncogenes. Our results point to a threefold to fourfold decrease in the binding affinity of both Kbu marks to these oncogenic targets in normal versus cancer cells. These results support Kbu direct targeting of genomic regions controlling growth and differentiation rather than inhibition of deacetylation and show that lncRNAs like *PRNCR1*, *PCAT1* and *CRAT37* are associated with Kbu binding in colorectal cells. It is generally thought that in cancer cells under aerobic metabolism, butyrate (and to some extent propionate) accumulates and acts as an HDAC inhibitor, leading to apoptosis^39^. Here, we have expanded on the role of SCFAs as unique regulatory elements and have shown the genome-wide localization of H3K18pr, H3K18bu, H4K12pr and H4K12bu in CRC cells and, in the case of Kbu, in mouse intestines as well.

Our data point to a mechanism involving dysregulation of a broad range of oncogenes such as *MYC* and tumour-suppressing genes such as *TGF-βR2* and *SMAD2/3*. Our integrated multiomics data show the unique role of propionate and butyrate as regulators of histone acyl lysine levels leading to increased chromatin accessibility. In cancer cells, this results in overexpression of already high levels of proto-oncogenes controlling growth and differentiation, which may ultimately lead to cell death, especially in instances of elevated butyrate levels. This model extends the existing repertoire of SCFAs as epigenetic regulators in addition to inhibitors of deacetylation. Regarding HDAC activity, especially in the case of propionate, we did not observe significant increases in acetylation in our CRC cell line. In addition, for each Kpr and Kbu ChIP-seq experiment, we used the corresponding Kac marks as controls to distinguish each histone mark’s unique targets under enrichment.

These data support a model that colonic SCFAs produced by microbial metabolism of fibre increase epithelial homeostatic gene expression pathways and impair carcinogenesis by direct histone modification. Given the rapid increase in CRCs, especially in younger-age populations recently, our results imply that dietary factors should be optimized to improve human health and diminish cancer onset. For example, the results raise the possibility of modulating histone PTMs with the use of dietary adjuvants or through the creation of synthetic acyl chains to more precisely tune colonic epithelial gene expression^40^.

In addition, modulation of microbial populations or microbial metabolism may lead to improved epithelial homeostasis by epigenetic remodelling from microbial-derived acyl intermediates. Finally, given the high concentrations of SCFAs in the colonic environment, our results raise the possibility that chromatin-embedded acylations could serve as a storage depot of acylations that may be removed and further metabolized or recycled when colonic SCFA levels drop as a result of fibre limitation or antibiotic usage that destroys microbial populations^41–44^. Taken together, our results highlight the crucial importance of understanding mechanisms of SCFA use and point towards ways of applying SCFA epigenetic modifications to improve human health.

## Methods

### Cell lines

Adherent SW480, CCD841 and CT26 cells were obtained from ATCC (SW480, no. CLL-228; CCD841 CoN, no. CRL-1790; CT26.WT, no. CRL-2638) and cultured in commercially grown EMEM medium containing 10% FBS and 1% penicillin–streptomycin or EMEM-glucose and 1% penicillin–streptomycin (ScienceCell) and passaged every 3–5 days at 70–75% confluence. CT26 cells were cultured in RPMI medium containing 10% FBS and 1% penicillin–streptomycin. Cells were treated with 0–10 mM NaPr and NaBu for 12 h, followed by cell counting and collecting. All cell lines were maintained at 37 °C (33 °C in the case of CCD841) in a humidified atmosphere containing 5% CO_2_. All cell lines were tested for mycoplasma contamination by the vendor and authenticated using STR profiling.

### Immunoblots

Histones were first acid-extracted as described below. Protein extracts were made in RIPA buffer and quantitated by BCA assay, then diluted to equal concentrations and mixed with 4× LDS sample buffer and sample reducing agent (Invitrogen). Polyacrylamide gel electrophoresis was performed on NuPAGE Novex gradient gels (Thermo Fisher) followed by wet transfer to nitrocellulose membranes. Blocking was briefly performed with 5% non-fat milk. The primary antibody was incubated overnight at 4 °C in 5% milk, followed by washing in PBST. Then, the HRP-conjugated secondary antibody (Cell Signaling) was applied at room temperature for 1 h, followed by additional washing. The signal was developed with ECL pico or femto kits (Thermo Fisher) and imaged using the ChemiDoc Imaging system (Bio-Rad).

### RNA-mediated interference and cDNAs

HAT1 shRNAs were purchased from OriGene with the following sequences:

A. GATGGCACTACTTTCTAGTATTTGAGAAG,
B. AAGGATGGAGCTACGCTCTTTGCGACCGT and
C. TCCTACAGTTCTTGATATTACAGCGGAAG.

The HAT1 cDNA was purchased from OriGene (cat. no. RC209571L1) and the 5′ end was extended by a Gibson assembly reaction to add the additional 255 nucleotides to obtain a full-length clone. Mutagenesis of this cDNA was performed with the QuikChange II Site-Directed Mutagenesis kit with the following primers to make the D276Q acetylation-dead form:

CAGTTCTTGATATTACAGCGCAAGATCCATCCAAAAGCTAT and

ATAGCTTTTGGATGGATCTTGCGCTGTAATATCAAGAACTG.

To mutate the shRNA-C target site, mutagenesis was performed with the following primers:

GGTCGCAAAGAGCGTAGCTCCGTCTTTGTTGTATTTCTCAAATACTAGAAAGTA

GTGCCATCTTTCATC and

GATGAAAGATGGCACTACTTTCTAGTATTTGAGAAATACAACAAAGACGGAGC

TACGCTCTTTGCGACC.

### Cell viability assays

Cell viability in the presence of increasing levels of NaPr and NaBu was performed using the CellTiter-Blue Cell Viability Assay (Promega). TSA was used as a negative control. The assay measures the ability of living cells to convert a redox dye (resazurin) into a fluorescent end product (resorufin). Nonviable cells do not generate a fluorescent signal. A total of 100 µl (or ~5,000 adherent cells) were plated in 96-well plates in EMEM containing 10% FBS and 1% penicillin–streptomycin. All cell lines were maintained at 37 °C in a humidified atmosphere containing 5% CO_2_. The medium was aspirated after 24 h and replaced with medium containing increasing levels of NaPr and NaBu (0–100 mM) and TSA (0–100 µM) in tenfold increments with three replicates per condition. After 72 h, 40 µl of CellTiter-Blue reagent was added to each well and cells were incubated for 4 h. Following incubation, fluorescence at 560/590 nm was measured using an Infinite M1000 Tecan i-control (v.1.10.4.0) plate reader.

### Histone acid extraction

SW480 and CCD841 colorectal cells were grown in EMEM containing 10% FBS and 1% penicillin–streptomycin. All cell lines were maintained at 37 °C. Before reaching confluence, cells were treated with increasing levels (0 mM, 0.1 mM, 1 mM and 10 mM) of ^13^C_3_-NaPr (Cambridge Biosciences, CLM-1865) for 12 h. Cells were collected and pelleted at 218*g* (RCF) for 5 min at 4 °C. Cells were resuspended in TEB buffer (PBS supplemented with 0.5% Triton X-100, 2 mM phenylmethyl sulfonyl fluoride, 0.02% NaN_3_) in 1 ml per 10^7^ cells. Cells were incubated on ice for 10 min with gentle stirring then centrifuged at 1,966*g* for 5 min at 4 °C. Pellets were resuspended in 200 µl of extraction buffer per 10^7^ cells. Cells were then incubated on ice for 30 min and centrifuged at 3,209*g* for 5 min at 4 °C. The supernatant was acetone-precipitated in 600 µl of acetone per 10^7^ cells and incubated overnight at −20 °C. Histones were then acid-extracted and acetone-precipitated. Pellets were air-dried and saved for downstream MS analysis or resuspended in 100 µl of H_2_O and diluted to 1 µg or 10 µg levels for immunoblot analysis. Protein concentration was determined using Pierce BCA Protein Assay Kits (Thermo Scientific, cat. no. 23225) with BSA as a standard.

### Mass spectrometric identification of histone Kpr

Histone extracts were purified and derivatized according to a previous publication^45^ and analysed by nano-capillary liquid chromatography triple quadrupole mass spectrometry (nLC-QqQ MS). The digested and derivatized histone peptides were diluted in 0.1% TFA and injected to nLC-QqQ MS (Dionex nanoLC and a Thermo Fisher Scientific TSQ Quantum). Peptides were first loaded into a trapping column (2 cm × 150 μm) and then separated with an analytical capillary column (10 cm × 75 μm). Both were packed with Magic C18 resin (Michrom). The chromatography gradient was achieved by increasing the percentage of buffer B from 2–35% at a flow rate of 0.35 μl min^−1^ (A, 0.1% formic acid in water; B, 0.1% formic acid in acetonitrile) over 40 min. The peptides were then introduced into QqQ MS by electrospray from an emitter with a 10 μm tip (New Objective) as they were eluted from the capillary column. The QqQ settings were as follows: collision gas pressure of 1.5 mTorr; Q1 peak width of 0.7 (FWHM); cycle time of 3.5 s; skimmer offset of 10 V; and electrospray voltage of 2.6 kV. Targeted histone PTM analysis was carried out using multiple reaction monitoring on a Thermo Scientific TSQ (triple-stage quadrupole) instrument. Only specific precursor peptides were fragmented and specific product ion intensities were measured. Chromatographic separation and MS-measured intensities of different forms of peptides were used to distinguish modifications of interest. Exogenously introduced lysine Kpr was reported as the ^13^C/^12^C heavy/light ratio.

### Determination of Acyl-CoA levels by LC–MS/MS and data analysis

Acetyl-CoA (≥93% purity by HPLC), propionyl-CoA (≥85% purity by HPLC) and butyryl-CoA (≥90% purity by HPLC) standards were purchased from Sigma-Aldrich. ^13^C_2_ acetyl-CoA (97% purity by HPLC), used as an internal standard, was purchased from Millipore-Sigma. Individual analytes and internal standard primary stock solutions (1 mg ml^−1^) were prepared separately in 10 mM ammonium acetate and water. The intermediate stock solution was prepared by mixing individual stock solutions of each analyte followed by dilution to yield a 200 µg ml^−1^ working solution. This working solution was serially diluted with 10 mM ammonium acetate in water to obtain a series of standard spiking solutions, which were used to generate the calibration curve. Calibration curves were prepared by spiking 10 µl of each standard working solution into 50 µl of homogenization buffer (methanol and water, 1:1 v/v) followed by the addition of 10 µl internal standard solution (1,000 ng ml^−1^ ^13^C_2_ acetyl-CoA). A calibration curve was prepared fresh with each set of samples. The calibration curve range for all analytes was 0.5–1,000 ng ml^−1^ using a 50 µl aliquot. All cell lines were maintained at 37 °C in EMEM containing 10% FBS and 1% penicillin–streptomycin as described previously. The medium was aspirated after 24 h and replaced with medium containing increasing levels of NaPr and NaBu (0–100 mM) in tenfold increments with three replicates per condition. Adherent cells were detached using a 0.25% (w/v) Trypsin-0.53 mM EDTA solution at 37 °C for 5 min. Cells were quenched in media and counted using an automated cell counter. The cells were resuspended in 2 ml and then transferred to 2 ml tubes, spun down again and aspirated, and the pellets were stored at −80°C. A 200 µl cold methanol and water (1:1 v/v) solution was added to each cell pellet. Samples were vortexed and sonicated twice for 2 min followed by centrifugation for 10 min at 2,229*g* at 4 °C. Then, 50 µl of the supernatant was transferred to a clean Eppendorf tube (1.7 ml). Next, 10 µl of internal standard solution was added to a 50 µl aliquot of supernatant followed by vortexing, 100 µl of an ice-cold solution of methanol was added to the sample and vortexed and the samples were stored at −20 °C for 1 h to facilitate protein precipitation. After centrifugation, the supernatant was transferred to a glass test tube and evaporated to dryness under nitrogen at 60 °C, reconstituted in 10 mM ammonium acetate buffer, sonicated, vortexed and transferred to an injection vial and analysed by LC–MS/MS. All analyses were carried out by positive electrospray LC–MS/MS using a Waters Acquity I-class UPLC system with Waters Xevo TQ-XS triple quadrupole mass spectrometer (RRID: SCR_018510). A Waters Atlantis T3 2.1×100 mm 3 µm particle size column (P/N1860203718) was operated at 40 °C at a flow rate of 0.2 ml min^−1^. Mobile phases consisted of 10 mM ammonium acetate in water (A) and acetonitrile (B). The elution profile consisted of a linear gradient of 0–25% B from 0–5 min, followed by a linear gradient of 25–95% B for 1 min, held at 95% for 2 min and equilibrated back to 0% B, for a total run time of 10 min. The injection volume was 10 µl. Selected reaction monitoring was used for quantification. The mass transitions of the analytes were as follows: acetyl-CoA: *m*/*z* 810.0 → 303.1 (quantifier) and *m*/*z* 810.0 → 135.9 (qualifier); propionyl-CoA: *m*/*z* 824.1 → 317.1 (quantifier) and *m*/*z* 824.1 → 135.9 (qualifier); butyryl-CoA: *m*/*z* 838.1 → 331.1 (quantifier) and *m*/*z* 838.1 → 427.9 (qualifier); for the internal standard: ^13^C_2_ acetyl-CoA *m*/*z* 812.0→ 305.0 (quantifier) and *m*/*z* 812.0 → 135.9 (qualifier). Quantitative analysis was done with TargetLynx quantification software (Waters) using an internal standard approach. Calibration curves were linear (*R* > 0.99) over the concentration range using a weighting factor of 1 / *X*^2^ (where *X* is the concentration). The back-calculated standard concentrations were ±15% from nominal values and ±20% at the lower limit of quantitation (0.5 ng ml^−1^).

### ChIP-seq and data analysis

For H3K18ac/pr/bu and H4K12ac/pr/bu ChIP-seq, cells were trypsinized and crosslinked with 1% formaldehyde (EMD Millipore) for 10 min at 20–22 °C. To quench the formaldehyde, 2 M glycine (Thermo Fisher Scientific) was added and incubated for 5 min at room temperature. Cells were washed twice with ice-cold PBS, snap-frozen and stored at −80 °C. For ChIP-DNA preparation, cells were thawed by adding PBS and incubated at 4 °C with rotation. Cells were treated with hypotonic buffer (20 mM HEPES pH 7.9, 10 mM KCl, 1 mM EDTA pH 8.0, 10% glycerol) for 10 min on ice in the presence of protease inhibitors (G6521, Promega). Nuclear pellets were resuspended in RIPA buffer (Millipore) and incubated for 30 min on ice. Chromatin corresponding to 10 million cells for histone modifications was sheared with SFX250 Sonifier (Branson) in three sets of three 30 s sonications, set to an intensity (output control) of 3.5. The lysates were transferred to Diagenode tubes and sonicated for 16 rounds of 30 s on and 30 s off, vortexing every fourth round. Protein G beads (80 µl) and lysates were washed with RIPA buffer and immunoprecipitated with antibodies targeting H3K18ac (Abcam, no. ab40888), H3K18pr (PTM Bio, no. PTM-213), H3K18bu (Abcam, no. ab241458), H4K12ac (Abcam, no. ab46983), H4K12pr (PTM Bio, no. PTM-209) and H4K12bu (Abcam, no. ab241120), 5 μg for each condition, at 4 °C overnight on a nutator. For the input sample, 200 μl of sheared nuclear lysate was removed and stored overnight at 4 °C. On the second day, supernatants containing ChIP-DNA and input were reverse-crosslinked by incubating overnight at 65 °C in 1% SDS and 1× TE buffer. On the third day, ChIP-DNA was treated with 250 μl 1× TE buffer containing 100 μg RNase A (QIAGEN) and 5.0 μl of 20 mg ml^−1^ proteinase K (Thermo Fisher Scientific) and then purified using QIAGEN QIAquick purification columns. The ChIP-DNA samples were end-repaired using End-It DNA End Repair Kit (Lucigen) and A-tailed using Klenow Fragment and dATP (New England Biolabs). Illumina TruSeq adaptors (Illumina) were ligated and libraries were size-selected (200−400 bp) by gel extraction before PCR amplification. The purified libraries were subjected to paired-end sequencing on the Illumina HiSeq 4000/NovaSeq 6000 SP to obtain an average of approximately 30−35 million uniquely mapped reads for each sample.

The resulting data were processed using the Kundaje Lab ChIP-seq ENCODE processing pipeline (v.2.2.2) (https://github.com/ENCODE-DCC/chip-seq-pipeline2). In brief, this pipeline takes FASTQ files for ChIP samples and input as controls, and it outputs peak calls (bound regions). Alignments to bound regions (GRCh38.p13) were performed using Bowtie2 (10.1186/gb-2009-10-3-r25) and peak calling was performed using MACS2 (10.1186/gb-2008-9-9-r137). Peak flies were analysed for differential binding using the R package DiffBind (v.2.4.8) to produce a count matrix with an FDR-adjusted *P* value cutoff of <0.05. Data visualization was performed in R as well as using the Integrative Genomics Viewer (http://www.broadinstitute.org/igv). Differential motif enrichment analysis in HOMER (http://homer.ucsd.edu/homer) was performed using the function findMotifsGenome with default parameters to search for motif enrichment in the fully accessible regions and control immunoprecipitation and/or input used as a background. Annotation was performed using R packages ChIPpeakAnno (v.3.32.0) and ChIPseeker (v.1.34.1). TSS distribution heatmaps and profiles were determined using the computeMatrix, plotHeatmap and plotProfile functions within deepTools (v.3.1.0) (https://deeptools.readthedocs.io/en/develop/index.html). Genomic coordinate overlaps were determined using the intersect ‘function’ in bedtools (v.2.31.0), whereby the original entry in one set was reported once if any overlaps were found in the second set, effectively reporting that at least one overlap was found (https://bedtools.readthedocs.io/en/latest/content/tools/intersect.html). GO analysis was performed using GREAT (http://great.stanford.edu/public/html/index.php) and ShinyGO (10.1093/bioinformatics/btz931). Images in KEGG pathway analysis were produced by PATHVIEW (https://bioconductor.org/packages/release/bioc/html/pathview.html).

### ATAC-seq and data analysis

ATAC-seq was performed using Omni-ATAC as previously described^46^. In brief, 50,000 viable cells were pelleted at 500*g* for 5 min at 4 °C. Cells were resuspended in 50 μl of ATAC-resuspension buffer containing 0.1% NP-40, 0.1% Tween-20 and 0.01% digitonin and mixed by pipetting. Following incubation on ice for 3 min, cells were washed with 1 ml cold RSB containing 0.1% Tween-20 but no NP-40 or digitonin. Nuclei were pelleted at 500*g* for 10 min at 4 °C. Cells were resuspended in 50 μl of transposition mix containing 25 µl 2× TD buffer, 2.5 µl transposase (Illumina Tagmented DNA enzyme and buffer kit, 20034210) (100 nM final), 16.5 µl PBS, 0.5 µl of 1% digitonin, 0.5 µl of 1% digitonin, 0.5 µl of 10% Tween-20 and 5 µl H_2_O). The final reaction was incubated at 37 °C for 30 min in a thermomixer with 1,000*g* mixing. Pre-amplified transposed fragments were cleaned using the Zymo DNA Clean and Concentrator-5 Kit (cat. no. D4013). DNA was eluted in 21 µl of elution buffer. Samples then underwent five cycles of PCR using NEBNext 2× MasterMix. Each reaction contained 2.5 µl of 25 µM i5 primer, 2.5 µl of 25 µM i7 primer, 25 µl of 2× NEBNext MasterMix and 20 µl of transposed or cleaned-up sample. Reactions were PCR-amplified (72 °C for 5 min, 98 °C for 30 s, followed by five cycles of 98 °C for 10 min, 63 °C for 30 s, 72 °C for 1 min; then held at 4 °C. Using 5 µl of pre-amplified mixture, 15 μl qPCR (Applied Biosystems, Quantstudio 6 Flex) was then performed to determine the additional number of cycles. The conditions for qPCR were as follows: 3.76 μl sterile H_2_O, 0.5 μl of 25 µM i5 primer, 25 µM of i7 primer, 0.24 μl of 25× SYBR gold (in dimethylsulfoxide), 5 μl of 2× NEBNext MasterMix and 5 μl of pre-amplified sample. Cycling conditions for qPCR were 98 °C for 30 s followed by 20 cycles of (98 °C for 10 s, 63 °C for 30 s and 72 °C for 1 min). qPCR amplification profiles were then manually assessed to determine the required number of additional cycles. Using the remainder of the pre-amplified DNA, two to three additional cycles were performed. Final PCR reactions were purified using Zymo DNA Clean and Concentrator-5 Kit (cat. no. D4013) and eluted in 20 μl of H_2_O. Amplified DNA library concentration was determined using a Qubit 4 Fluorometer (Invitrogen). Library quality and size were assessed on an Agilent Bioanalyzer 2100 system using a high-sensitivity DNA kit. Multiplexed libraries were paired-end sequenced on the Illumina HiSeq 4000/NovaSeq 6000 SP to obtain an average of approximately 50 million uniquely mapped reads per sample. The resulting data were processed using the Kundaje Lab ENCODE ATAC-seq processing pipeline (v.2.2.3) (https://github.com/ENCODE-DCC/atac-seq-pipeline). In brief, this pipeline takes FASTQ files as input and outputs peak calls (accessible regions). All annotations were carried out against the GENCODE annotation database for human (GRCh38.p13) and mouse (GRCm38.p4) genomes. Alignments to accessible regions were performed using Bowtie2 (10.1186/gb-2009-10-3-r25) and peak calling was performed using MACS2 (10.1186/gb-2008-9-9-r137). Peak flies were analysed for differential accessibility using the R package DiffBind (v.2.4.8) to produce a counts matrix with an FDR-adjusted *P* value cutoff of <0.05. Venn diagrams were generated by https://bioinformatics.psb.ugent.be/webtools/Venn.

### CUT&Tag and data analysis

CUT&Tag analysis of NaBu-treated CCD841 cells and mouse intestinal samples was performed using CUT&Tag-IT assay kits (Active Motif, nos. 53160 and 53170). Approximately 30 mg of tissue was homogenized per reaction by first chopping frozen tissue into smaller pieces in a Petri dish with a razor blade on dry ice. Then, 1 ml of CUT&Tag-IT Lysis Buffer (50 mM Tris, pH 8.0, 10 mM EDTA, 0.4% w/v SDS and 0.1% protease inhibitor cocktail) was added per 10 mg of tissue and samples were further cut and minced in bulk. Samples were then Dounced 20 times with a loose pestle and 10 times with a tight pestle. Samples were then filtered with a 40 µM strainer in 15 ml conical tubes. Samples were centrifuged at 500*g* for 5 min at 4^o^C. Samples of nuclei suspension were counted and normalized to ~500,000 nuclei per reaction. CCD841 cells (~500,000 per reaction) treated with NaBu for 12 h were collected by centrifugation at 600*g* at room temperature. Both CCD841 cells and mouse tissue extracts were washed with 1× wash buffer (1 M HEPES, pH 7.5, 1.5 ml of 5 M NaCl, 12.5 μl of 2 M spermidine) containing protease inhibitor cocktail (10 μl per 1 ml of wash buffer) and resuspended in Concanavalin A Beads slurry in a 1× binding buffer (1 M HEPES, pH 7.5, 100 μl of 1 M KCl, 10 μl of 1 M CaCl_2_ and 10 μl of 1 M MnCl_2_). Cells and beads were incubated on an end-over-end rotator for 10 min. Cells were resuspended in ice-cold antibody buffer containing 2 ml dig-wash buffer (5% digitonin with 40 ml of 1× wash buffer with PIC) mixed with 8 μl of 0.5 M EDTA and 6.7 µl of 30% BSA not to exceed 500,000 cells per 50 μl reaction volume. Then, 5 μg of undiluted primary antibody was added to each sample for an overnight incubation at 4 °C with orbital mixing. Next, 100 μl of guinea pig anti-rabbit secondary antibody diluted 1:100 in dig-wash buffer was added to each reaction and the samples were incubated on an orbital rotator for 60 min at room temperature. Then, 100 μl of 1:100 diluted CUT&Tag-IT Assembled pA-Tn5 Transposomes in dig-300 buffer (1 M HEPES, pH 7.5, 3 ml of 5 M NaCl and 12.5 μl of 2 M spermidine with 5% digitonin and 0.01% protease inhibitor cocktail) was added to each sample and reactions were incubated on an orbital rotator for 60 min at room temperature. Following three washes with dig-300 buffer, 125 μl of tagmentation buffer (5 ml dig-300 buffer and 50 µl of 1 M MgCl_2_) was added to each sample and reactions were incubated at 37 °C for 1 h. To stop the tagmentation and solubilize DNA fragments, each sample received 4.2 μl of 0.5 M EDTA, 1.25 μl of 10% SDS and 1.1 μl of proteinase K (10 mg ml^−1^). Samples were mixed and incubated at 55 °C for 60 min. Following washing, samples were centrifuged at 17,000*g* for 2 min. PCR amplification was performed by adding 30 μl of tagmented DNA from each reaction to 1 µl of dNTPs (10 mM), 0.5 μl of NEBNext Q5 High-Fidelity DNA Polymerase, 10 μl of 5× Q5 reaction buffer, 3.5 μl of nuclease-free H_2_O and 2.5 μl of unique combinations of Nextera i5 and i7 indexing primers to a total of 50 μl reaction. PCR was performed using the following programme on a thermal cycler with a heated lid: cycle 1, 72 °C for 5 min (gap filling); cycle 2, 98 °C for 30 s; cycle 3, 98 °C for 10 s; cycle 4, 63 °C for 10 s. Cycles 3 and 4 were then repeated 14 times, held at 72 °C for 1 min and then held at 10 °C. PCR reactions were purified using Zymo DNA Clean and Concentrator-5 Kit (cat. no. D4013) and eluted in 20 μl of nuclease-free H_2_O. Amplified DNA library concentration was determined using a Qubit 4 Fluorometer (Invitrogen). Library quality and size were assessed on an Agilent Bioanalyzer 2100 system using a high-sensitivity DNA kit. Multiplexed libraries were paired-end sequenced on the Illumina HiSeq 4000/NovaSeq 6000 SP to obtain an average of approximately 50 million uniquely mapped reads per sample. The resulting data were processed using the Kundaje Lab ENCODE ATAC-seq processing pipeline (v.2.2.3) (https://github.com/ENCODE-DCC/atac-seq-pipeline). In brief, this pipeline takes FASTQ files as input and outputs peak calls (accessible regions). All annotations were carried out against the GENCODE annotation database for human (GRCh38.p13) and mouse (GRCm38.p4) genomes. Alignments to accessible regions were performed using Bowtie2 (10.1186/gb-2009-10-3-r25) and peak calling was performed using MACS2 (10.1186/gb-2008-9-9-r137). Peak flies were analysed for differential accessibility using the R package DiffBind (v.2.4.8) to produce a counts matrix with an FDR-adjusted *P* value cutoff of <0.05.

### RNA-seq and data analysis

RNA was extracted using the RNeasy Mini Kit (QIAGEN, no. 74136). Libraries were prepared using the NEBNext Ultra II RNA library prep kit for Illumina (no. E7770) and the rRNA depletion kit (no. E6310). Paired-end sequencing on the Illumina NovaSeq 6000 SP yielded an average of approximately 20 million uniquely mapped reads per sample for mRNA-seq. The resulting data were aligned to the human genome (GRCh38.p13) by STAR (v.2.5.4b) (https://github.com/alexdobin/STAR). The aligned transcripts were quantitated based on features in the GENCODE annotation database (GRCh38.p13) by RSEM (v.1.3.1) (http://deweylab.biostat.wisc.edu/rsem). Differentially expressed genes were detected using the R package DESeq2 (v.1.20.0) with an FDR-adjusted *P* value cutoff of <0.05 (https://bioconductor.org/packages/release/bioc/html/DESeq2.html). Venn diagrams were generated by https://bioinformatics.psb.ugent.be/webtools/Venn.

### Animal handling and preparation of intestinal tissue samples

All experiments were approved by and conducted in strict accordance with Stanford University’s Administrative Panel on Laboratory Animal Care (APLAC 34017). Male CETP-ApoB-100 transgenic mice were procured from Taconic Biosciences. All animals were housed in a temperature-controlled, specific-pathogen-free environment, with a 12-h light, 12-h dark cycle, temperature of 24 ± 1 °C and humidity ranging between 40% and 60%. Food and water were available ad libitum. Mice were assigned randomly to two dietary groups: a low-fat control diet (10% fat, TD 08485, Envigo Teklad) or a high-fat, high-sucrose diet (HFS; 42% fat, TD 88137, Envigo Teklad). The HFS diet was selected to mimic the Western diet, aiming to characterize and augment atherosclerosis development in the ApoE-deficient transgenic mouse. Over 4 weeks, both groups were subjected to their respective diets. Following the initial 4-week duration, the control group continued on their diet for an additional 4 weeks. By contrast, the HFS group received supplementation of 5% arabinoxylan (5% w/w of total HFS, blended in-house with their powdered HFS diet) for an additional 4 weeks. At the conclusion of this second 4-week period, all mice underwent a 6 h fast and were then anaesthetized with isoflurane and subsequently killed by cervical dislocation. The large intestine was dissected, weighed, flash-frozen in liquid nitrogen and subsequently stored at −80 °C until analysis.

There was no randomization in the organization of the experimental conditions or stimulus presentation. Data collection and analysis were not performed blind to the conditions of the experiments. No animals or data points were excluded from the analyses. No statistical methods were used to pre-determine sample sizes, but our sample sizes are similar to those reported in a previous publication^47^. The data met the assumptions of the statistical tests used, including normality and equal variances, which were formally tested by running diagnostics and visualizations such as residual, homoscedasticity and QQ plots using Brown–Forsythe and Shapiro–Wilk tests.

### Reporting summary

Further information on research design is available in the Nature Portfolio Reporting Summary linked to this article.

## Supplementary information

Supplementary Information Supplementary Figs. 1–8.

Reporting Summary

## Source data

Source Data Fig. 1 Statistical Source Data, Excel file with individual tabs for each experiment’s processed data.

Source Data Extended Data Figs. 1–9 Unprocessed western blots and/or gels.

## Data Availability

ChIP-seq, ATAC-seq and CUT&Tag raw data and differential peak call files have been uploaded to the Gene Expression Omnibus under accession numbers GSE252649, GSE252652 and GSE252754, respectively. RNA-seq data have been deposited in the Gene Expression Omnibus under accession number GSE252753. GSE252653 is the reference for the series of four datasets. Source data are provided with this paper.
