# Supplementary material for: Short-chain fatty acid metabolites propionate and butyrate are unique epigenetic regulatory elements linking diet, metabolism and gene expression
Source: Nat Metab. 2025 Jan 9;7(1):196–211. doi: 10.1038/s42255-024-01191-9 (PMC11774759; doi:10.1038/s42255-024-01191-9)
Supplement: Supplementary file 2 — Reporting Summary [file 42255_2024_1191_MOESM2_ESM.pdf]

nature portfolio

Corresponding author(s): Michael Snyder

Last updated by author(s): 11/10/2024

Reporting Summary

Nature Portfolio wishes to improve the reproducibility of the work that we publish. This form provides structure for consistency and transparency in reporting. For further information on Nature Portfolio policies, see our Editorial Policies and the Editorial Policy Checklist.

Please do not complete any field with "not applicable" or n/a. Refer to the help text for what text to use if an item is not relevant to your study.
For final submission: please carefully check your responses for accuracy; you will not be able to make changes later.

Statistics

For all statistical analyses, confirm that the following items are present in the figure legend, table legend, main text, or Methods section.

n/a	Confirmed	
☐	☑	The exact sample size (n) for each experimental group/condition, given as a discrete number and unit of measurement
☐	☑	A statement on whether measurements were taken from distinct samples or whether the same sample was measured repeatedly
☐	☑	The statistical test(s) used AND whether they are one- or two-sided<br>*Only common tests should be described solely by name; describe more complex techniques in the Methods section.*
☐	☑	A description of all covariates tested
☐	☑	A description of any assumptions or corrections, such as tests of normality and adjustment for multiple comparisons
☐	☑	A full description of the statistical parameters including central tendency (e.g. means) or other basic estimates (e.g. regression coefficient) AND variation (e.g. standard deviation) or associated estimates of uncertainty (e.g. confidence intervals)
☐	☑	For null hypothesis testing, the test statistic (e.g. F, t, r) with confidence intervals, effect sizes, degrees of freedom and P value noted<br>*Give P values as exact values whenever suitable.*
☑	☐	For Bayesian analysis, information on the choice of priors and Markov chain Monte Carlo settings
☑	☐	For hierarchical and complex designs, identification of the appropriate level for tests and full reporting of outcomes
☑	☐	Estimates of effect sizes (e.g. Cohen's d, Pearson's r), indicating how they were calculated

Our web collection on statistics for biologists contains articles on many of the points above.

Software and code

Policy information about availability of computer code

Data collection	SEQUEST in ProteomeDiscoverer v2.1, SGDC Data Transfer Tool vl.6.0, TargetLynx quantification software (Waters Corp.) for acyl-CoA quantitative analysis.
Data analysis	ENCODE ChIP-seq (v2.2.2) and ATAC-seq pipelines (v2.2.3), HOMER (v5.1), deeptools (v3.1.0), bedtools (v2.31.0), STAR (v2.5.3a), rsem (v.1.2.30), R (v3.6), DESeq2 (v1.20.0), DiffBind (v.2.4.8), GREAT (v4.0.4), ShinyGO (v0.741).

For manuscripts utilizing custom algorithms or software that are central to the research but not yet described in published literature, software must be made available to editors and reviewers. We strongly encourage code deposition in a community repository (e.g. GitHub). See the Nature Portfolio guidelines for submitting code & software for further information.

Data

Policy information about availability of data

All manuscripts must include a data availability statement. This statement should provide the following information, where applicable:

ChIP-seq, ATAC-seq and CUT&Tag raw data and differential peak call files have been uploaded to GEO with accession numbers GSE252649, GSE252652 and GSE252754, respectively. RNA-seq data are deposited in GEO with accession number GSE252753. GSE252653 is the reference for the Series of four data sets (https://www.ncbi.nlm.nih.gov/geo/query/acc.cgi?acc=GSE252653). All annotations have been carried out against GENCODE annotation database for human (GRCh38.p13) and mouse (GRCm38.p4) genomes.

- Accession codes, unique identifiers, or web links for publicly available datasets
- A description of any restrictions on data availability
- For clinical datasets or third party data, please ensure that the statement adheres to our policy

Research involving human participants, their data, or biological material

Policy information about studies with human participants or human data. See also policy information about sex, gender (identity/presentation), and sexual orientation and race, ethnicity and racism.

Reporting on sex and gender	not applicable
Reporting on race, ethnicity, or other socially relevant groupings	not applicable
Population characteristics	not applicable
Recruitment	not applicable
Ethics oversight	not applicable

Note that full information on the approval of the study protocol must also be provided in the manuscript.

Field-specific reporting

Please select the one below that is the best fit for your research. If you are not sure, read the appropriate sections before making your selection.

- [x] Life sciences
- [ ] Behavioural & social sciences
- [ ] Ecological, evolutionary & environmental sciences

For a reference copy of the document with all sections, see nature.com/documents/nr-reporting-summary-flat.pdf

Life sciences study design

All studies must disclose on these points even when the disclosure is negative.

Sample size	All experiments had on average 3-4 technical replicates per condition. In the mouse fiber study, each experimental group (normal chow vs 5% arabinoxylan) had three biological replicates per condition.
Data exclusions	No data were excluded from calculations.
Replication	All ChIP and ATAC samples passed the ENCODE guidelines for reproducibility (self-consistency ratio < 2). Proteomics and metabolomics data had three technical replicates and the replicates were successful as the average Pearson correlation across replicates was 0.70. These samples were successful replicates as features met strict FDR criteria across various comparisons and the main conclusions were possible with the given set of technical replicates for cells, and biological replicates for tissue samples.
Randomization	Randomization was not relevant to this study. All sequencing methods were conducted such that all samples were pooled together. All samples were used in the study and were assigned to their designated group: treated vs untreated for cells, or HFS diet vs HFS + 5% arabinoxylan fiber diet for mice.
Blinding	Blinding is not relevant to our study - we did not require separate experimental groups.

Behavioural & social sciences study design

All studies must disclose

Study description	
Research sample	
Sampling strategy	
Data collection	
Timing	
Data exclusions	
Non-participation	
Randomization	

Ecological, evolutionary & environmental sciences study design

All studies must disclose on these points even when the disclosure is negative.

Study description	
Research sample	
Sampling strategy	
Data collection	
Timing and spatial scale	
Data exclusions	
Reproducibility	
Randomization	
Blinding	

Did the study involve field work? ☐ Yes ☑ No

Field work, collection and transport

Field conditions	
Location	
Access & import/export	
Disturbance	

Reporting for specific materials, systems and methods

We require information from authors about some types of materials, experimental systems and methods used in many studies. Here, indicate whether each material, system or method listed is relevant to your study. If you are not sure if a list item applies to your research, read the appropriate section before selecting a response.

Materials & experimental systems

n/a	Involved in the study	
☐	☑	Antibodies
☐	☑	Eukaryotic cell lines
☑	☐	Palaeontology and archaeology
☐	☑	Animals and other organisms
☑	☐	Clinical data
☑	☐	Dual use research of concern
☑	☐	Plants

Methods

n/a	Involved in the study	
☐	☑	ChIP-seq
☐	☐	Flow cytometry
☐	☐	MRI-based neuroimaging

Antibodies

Antibodies used	Antibodies were purchased from Abcam and PTM-Bio.H3K18ac (Abcam #ab40888), H3K18pr (PTM Bio #PTM-213), H3K18bu (Abcam #ab241458), H4K12ac (Abcam #ab46983), H4K12pr (PTM Bio #PTM-209), H4K12bu (Abcam #ab241120).
Validation	Antibodies were validated for specificity for Western blot and ChIP-seq applications by the manufacturers (abcam and PTM-Bio), using dot blots, Western blots, Immunofluorescence and ChIP, as indicated on manufacturers' websites.

Eukaryotic cell lines

Policy information about cell lines and Sex and Gender in Research

Cell line source(s)	Adherent SW480, CCD841 and CT26 cells were obtained from ATCC (SW480 #CLL-228, CCD841 CoN, #CRL-1790, CT26.WT #CRL-2638).
Authentication	All cell lines were authenticated by ATCC using STR profiling.
Mycoplasma contamination	Each cell line was mycoplasma contamination tested by ATCC. No mycoplasma contamination was detected.
Commonly misidentified lines (See ICLAC register)	No commonly misidentified cell lines were used.

Palaeontology and Archaeology

Specimen provenance	
Specimen deposition	
Dating methods	

☐ Tick this box to confirm that the raw and calibrated dates are available in the paper or in Supplementary Information.

Ethics oversight	

Note that full information on the approval of the study protocol must also be provided in the manuscript.

Animals and other research organisms

Policy information about studies involving animals; ARRIVE guidelines recommended for reporting animal research, and Sex and Gender in Research

Laboratory animals	8-week old male CETP-ApoB-100 transgenic mice from Taconic Biosciences (New York, USA) were used in this study.
Wild animals	No wild animals were used in this study.
Reporting on sex	Only male mice were used in this study.
Field-collected samples	No field-collected samples were used in this study.
Ethics oversight	All experiments were approved by and conducted in strict accordance with Stanford University's Administrative Panel on Laboratory Animal Care (APLAC 34017).

Note that full information on the approval of the study protocol must also be provided in the manuscript.

Clinical data

Clinical trial registration	
Study protocol	
Data collection	
Outcomes	

Policy information about clinical studies

All manuscripts should comply with the ICMJE guidelines for publication of clinical research and a completed CONSORT checklist must be included with all submissions.

Dual use research of concern

Policy information about dual use research of concern

Hazards

Could the accidental, deliberate or reckless misuse of agents or technologies generated in the work, or the application of information presented in the manuscript, pose a threat to:

No	Yes	
☑	☐	Public health
☑	☐	National security
☑	☐	Crops and/or livestock
☑	☐	Ecosystems
☑	☐	Any other significant area

Experiments of concern

Does the work involve any of these experiments of concern:

No	Yes	
☑	☐	Demonstrate how to render a vaccine ineffective
☑	☐	Confer resistance to therapeutically useful antibiotics or antiviral agents
☑	☐	Enhance the virulence of a pathogen or render a nonpathogen virulent
☑	☐	Increase transmissibility of a pathogen
☑	☐	Alter the host range of a pathogen
☑	☐	Enable evasion of diagnostic/detection modalities
☑	☐	Enable the weaponization of a biological agent or toxin
☑	☐	Any other potentially harmful combination of experiments and agents

Plants

Seed stocks	
Novel plant genotypes	
Authentication	

ChIP-seq

Data deposition

☑ Confirm that both raw and final processed data have been deposited in a public database such as GEO.

☑ Confirm that you have deposited or provided access to graph files (e.g. BED files) for the called peaks.

Data access links<br>*May remain private before publication.*	GEO accession GSE252653 (https://www.ncbi.nlm.nih.gov/geo/query/acc.cgi?acc=GSE252653) is the reference for the Series of data sets.
Files in database submission	fastq, bam, narrowPeak
Genome browser session<br>(e.g. UCSC)	No longer applicable.

Methodology

Replicates	All ChIP-seq experiments had on average 2-4 technical replicates plus input.
Sequencing depth	An average of approximately 30 - 35 million uniquely mapped reads for each sample.
Antibodies	Anti-H3K18ac, Anti-H3K18pr, Anti-H3K18bu, Anti-H4K12ac, Anti-H4K12pr, Anti-H4K12bu.
Peak calling parameters	macs2 callpeak -t </> -g hs -f BAM --outdir </> -n </> -q 0.05 --nomodel --extsize "size calculated on the strand cross correlation analysis" (ignored for PE reads)
Data quality	Phred quality score of 30 (Q30) was used as a base call cutoff. The q-value (minimum FDR) cutoff to call significant regions was set to 0.05.

Software	bowtie2, cutadapt, samtools, macs2, ucsc_tools

Flow Cytometry

Plots

Confirm that:

- ☐ The axis labels state the marker and fluorochrome used (e.g. CD4-FITC).
- ☐ The axis scales are clearly visible. Include numbers along axes only for bottom left plot of group (a 'group' is an analysis of identical markers).
- ☐ All plots are contour plots with outliers or pseudocolor plots.
- ☐ A numerical value for number of cells or percentage (with statistics) is provided.

Methodology

Sample preparation	
Instrument	
Software	
Cell population abundance	
Gating strategy	

☐ Tick this box to confirm that a figure exemplifying the gating strategy is provided in the Supplementary Information.

Magnetic resonance imaging

Experimental design

Design type	
Design specifications	
Behavioral performance measures	
Imaging type(s)	
Field strength	
Sequence & imaging parameters	
Area of acquisition	

Diffusion MRI ☐ Used ☐ Not used

Preprocessing

Preprocessing software	
Normalization	
Normalization template	
Noise and artifact removal	
Volume censoring	

Statistical modeling & inference

Model type and settings	
Effect(s) tested	

Specify type of analysis: ☐ Whole brain ☐ ROI-based ☐ Both

Statistic type for inference

(See Eklund et al. 2016)

Correction

Models & analysis

n/a	Involved in the study
☐	☐ Functional and/or effective connectivity
☐	☐ Graph analysis
☐	☐ Multivariate modeling or predictive analysis

Functional and/or effective connectivity

Graph analysis

Multivariate modeling and predictive analysis

This checklist template is licensed under a Creative Commons Attribution 4.0 International License, which permits use, sharing, adaptation, distribution and reproduction in any medium or format, as long as you give appropriate credit to the original author(s) and the source, provide a link to the Creative Commons license, and indicate if changes were made. The images or other third party material in this article are included in the article's Creative Commons license, unless indicated otherwise in a credit line to the material. If material is not included in the article's Creative Commons license and your intended use is not permitted by statutory regulation or exceeds the permitted use, you will need to obtain permission directly from the copyright holder. To view a copy of this license, visit http://creativecommons.org/licenses/by/4.0/
