# Supplementary material for: Short-chain fatty acid metabolites propionate and butyrate are unique epigenetic regulatory elements linking diet, metabolism and gene expression
Source: Nat Metab. 2025 Jan 9;7(1):196–211. doi: 10.1038/s42255-024-01191-9 (PMC11774759; doi:10.1038/s42255-024-01191-9)
Supplement: Supplementary file 4 — Unprocessed western blots and/or gels. [file 42255_2024_1191_MOESM4_ESM.pdf]

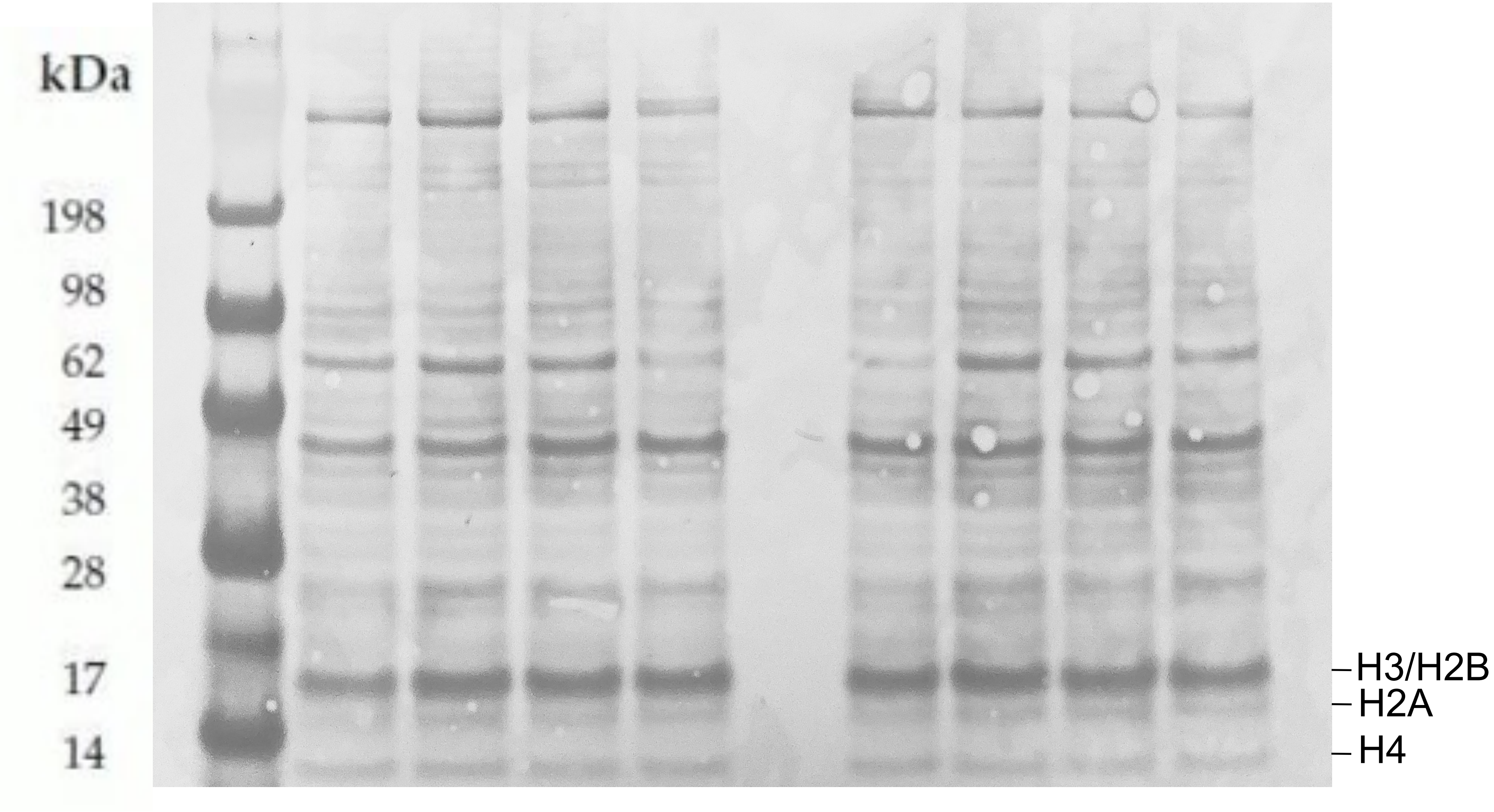

Source Data Extended Data Fig. 1 | Protein gel showing histones H2A/H2B, H3 and H4

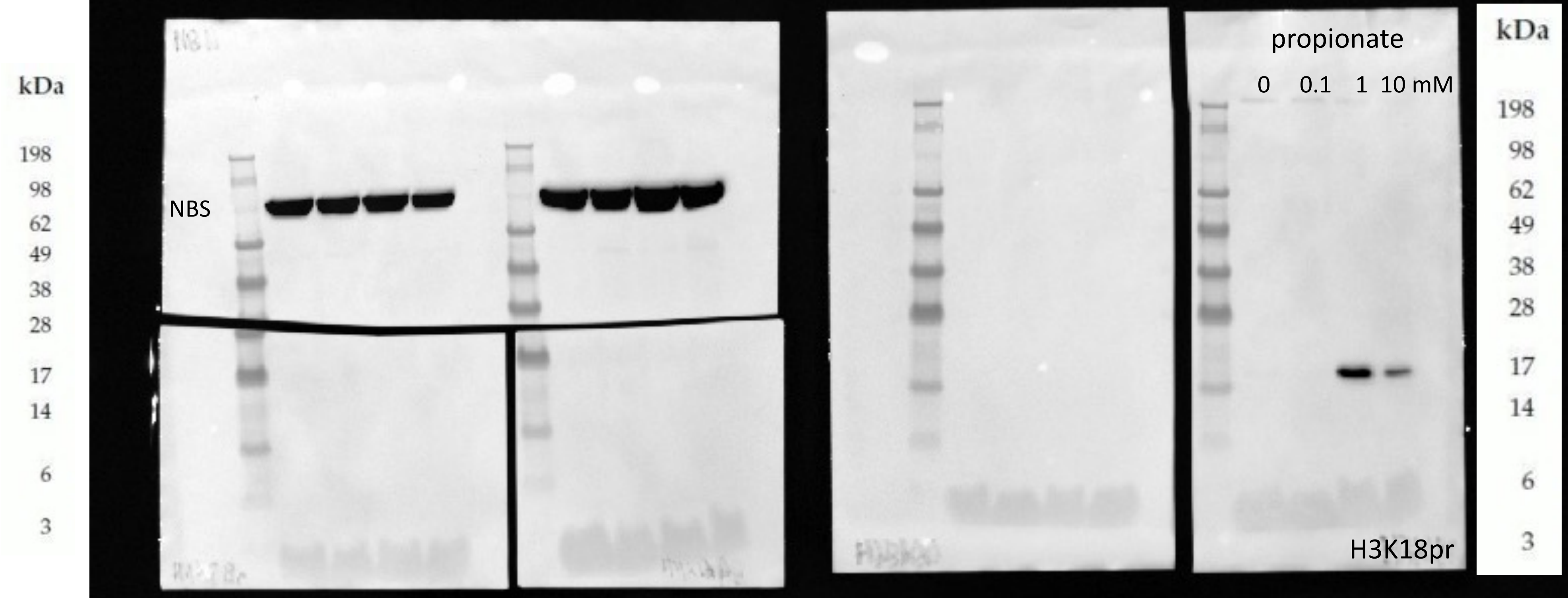

Source Data Extended Data Fig. 2 | H3K18pr immunoblot showing signal at 1 and 10mM propionate treatment

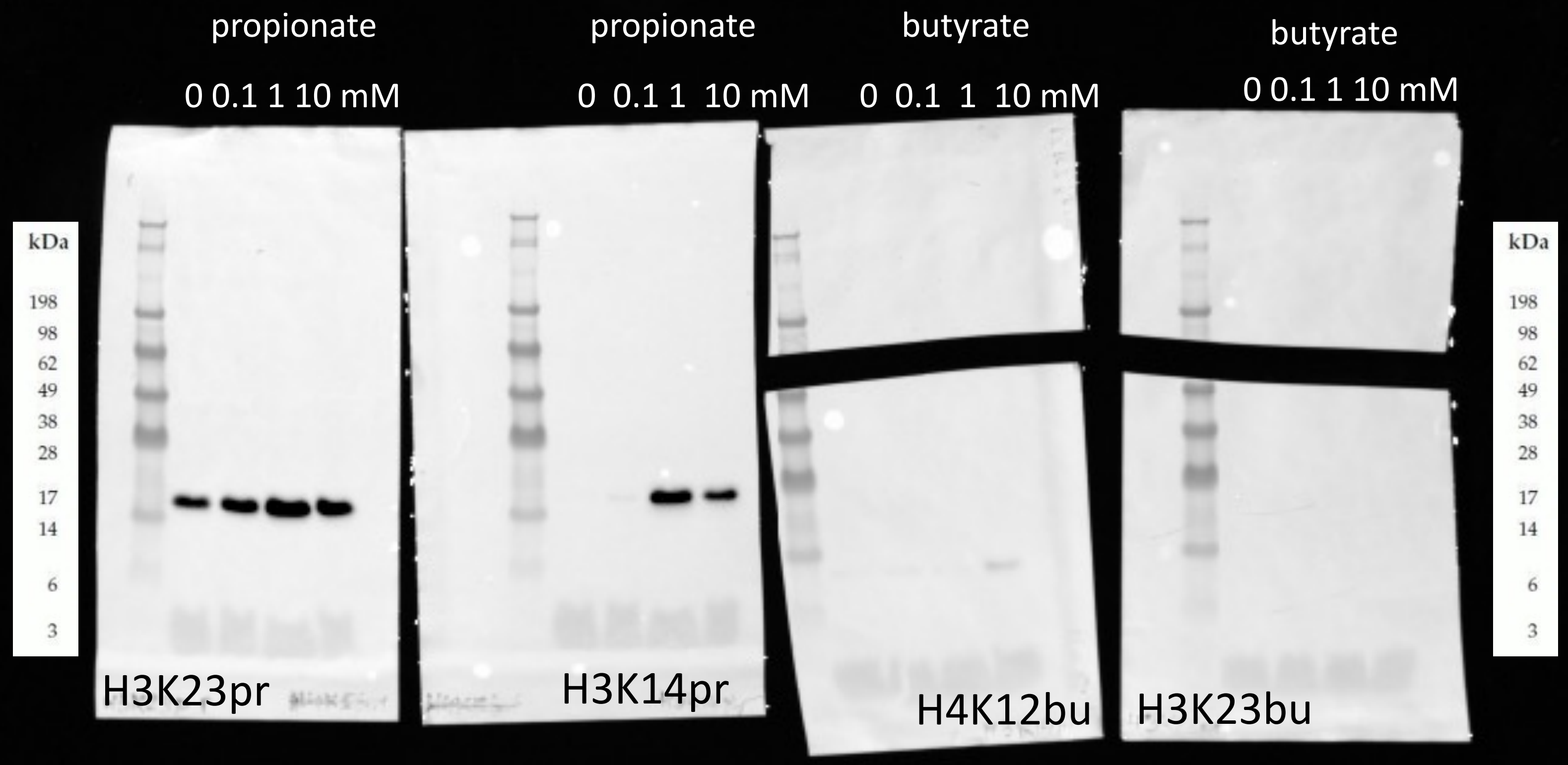

Source Data Extended Data Fig. 3 | H3K23pr, H3K14pr, H4K12bu, H3K23bu immunoblots showing signals at 1 and 10mM propionate and butyrate treatments.

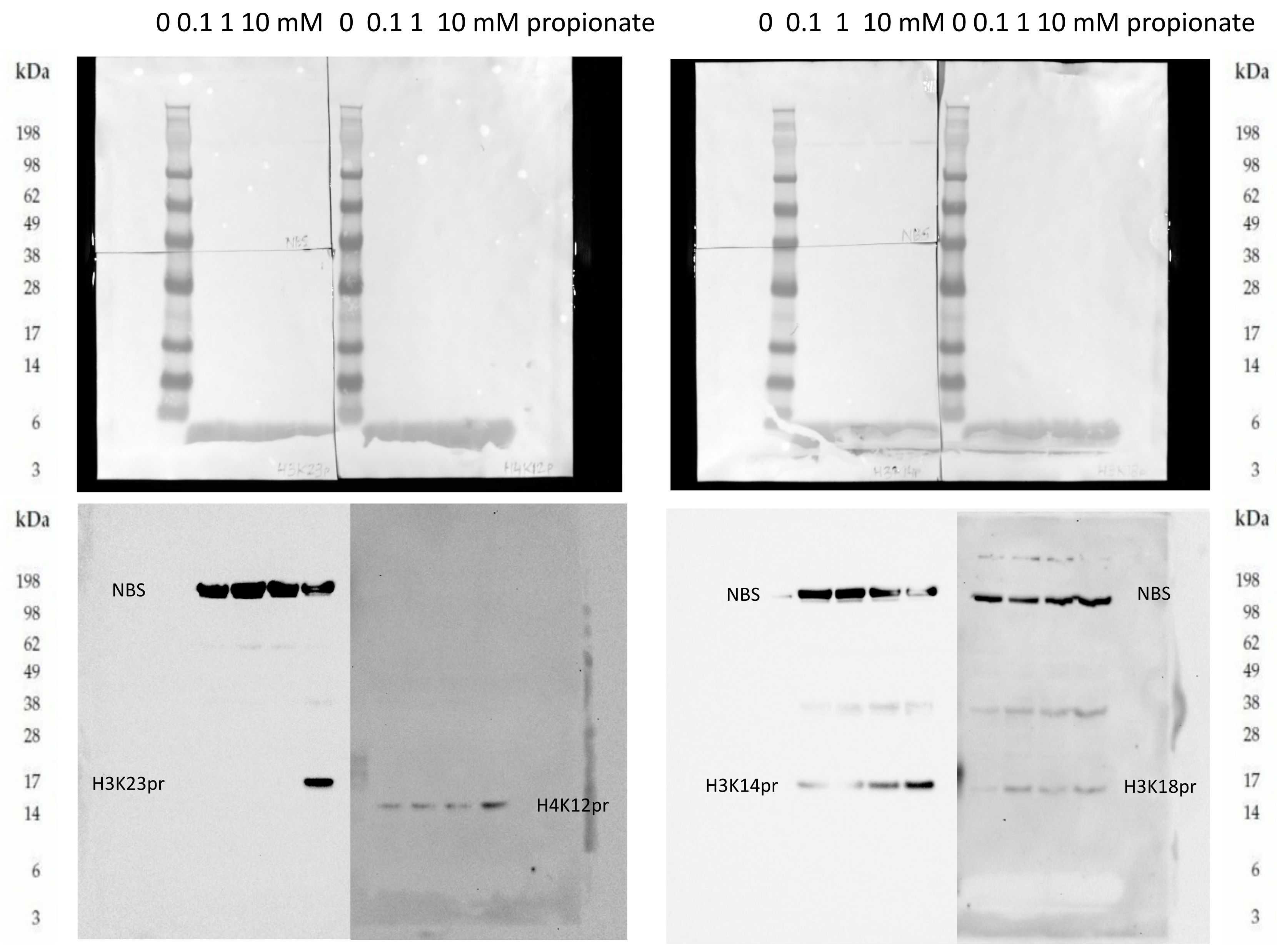

Source Data Extended Data Fig. 4 | H3K23pr, H4K12pr, H3K14pr, H3K18pr immunoblots showing signals at 1 and 10mM propionate treatments.

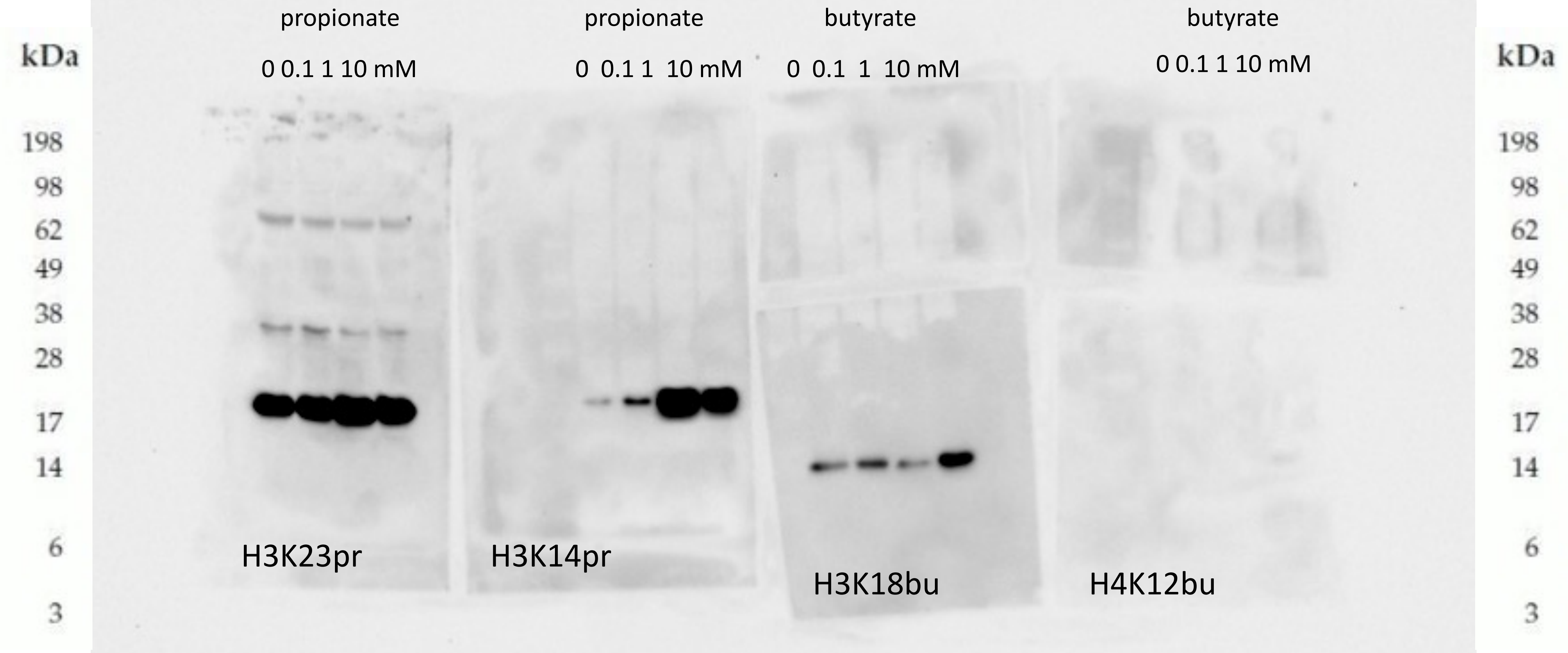

Source Data Extended Data Fig. 5 | H3K23pr, H3K14pr, H3K18bu and H4K12bu immunoblots showing signals at 1 and 10mM propionate and butyrate treatments.

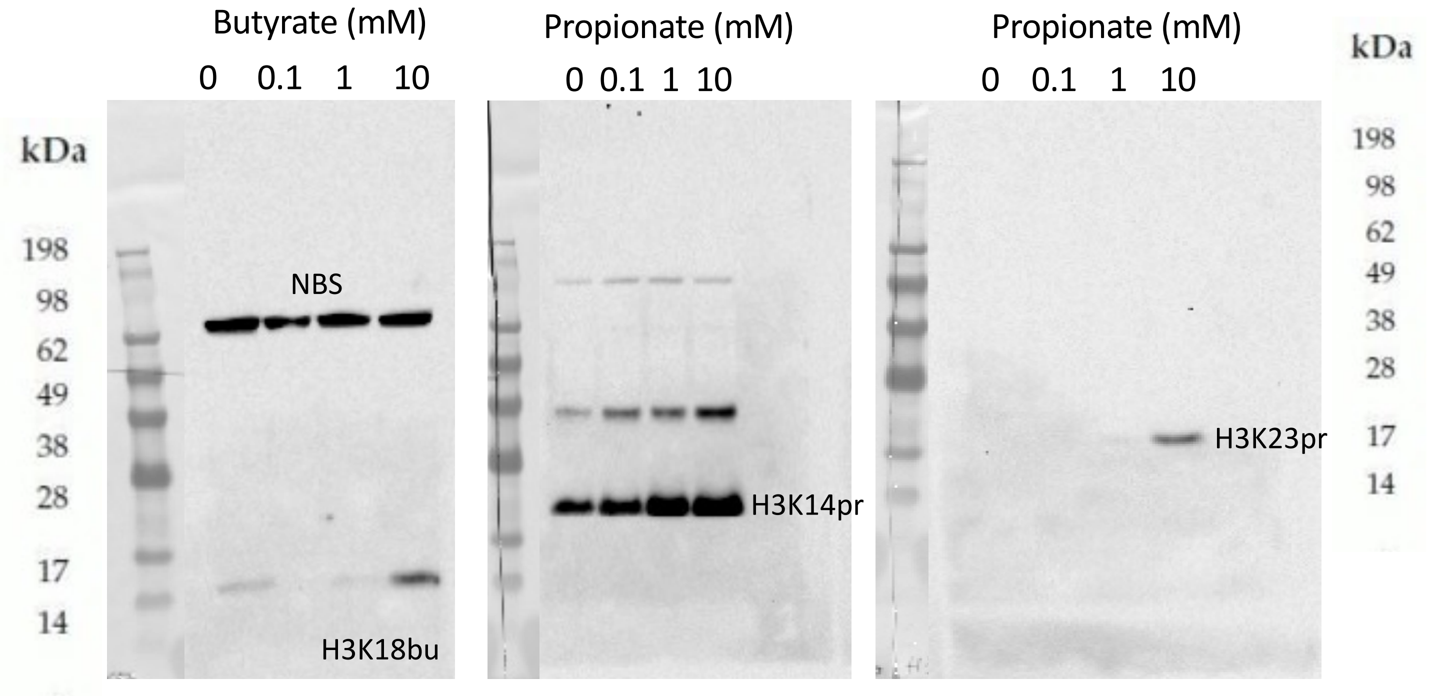

Source Data Extended Data Fig. 6 | H3K18bu, H3K14pr, H4K12bu, H3K23pr immunoblots showing signals at 1 and 10mM propionate and butyrate treatments.

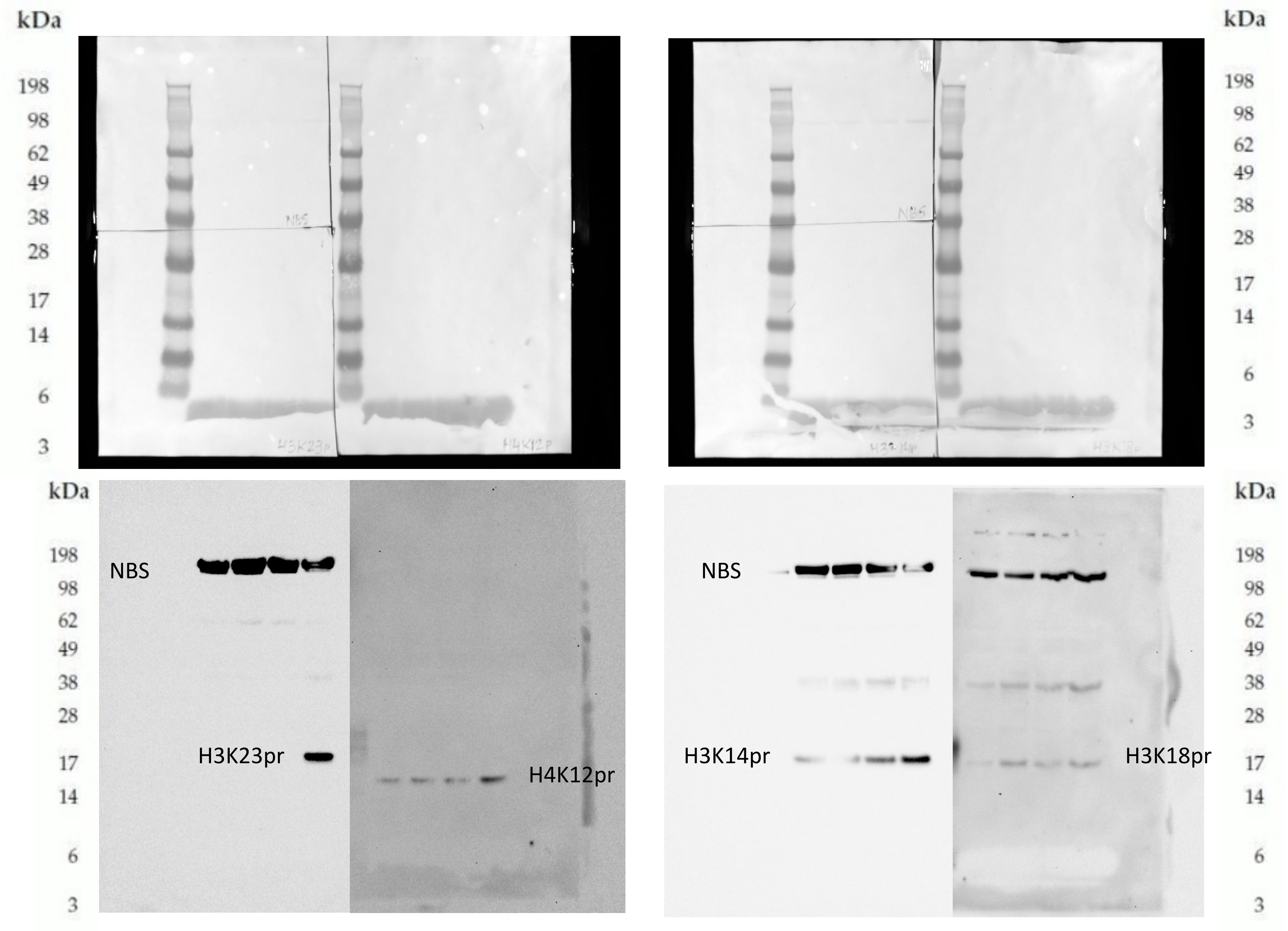

Source Data Extended Data Fig. 7 | H3K23pr, H4K12pr, H3K14pr, H3K18pr immunoblots showing signals at 1 and 10mM propionate and butyrate treatments.

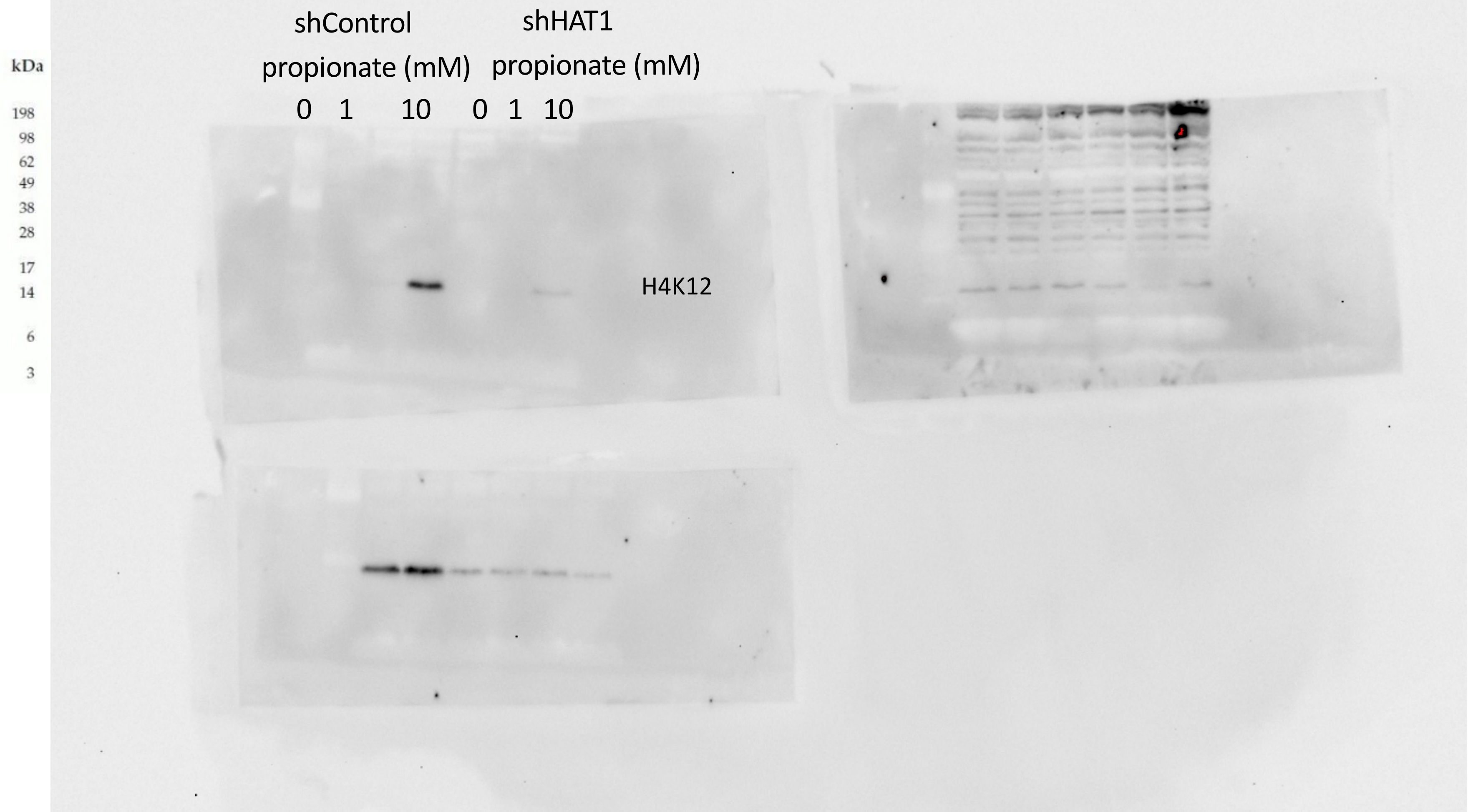

Source Data Extended Data Fig. 8 | Depletion of HAT1 diminishes incorporation of propionate into the H4 lysine 12 site.

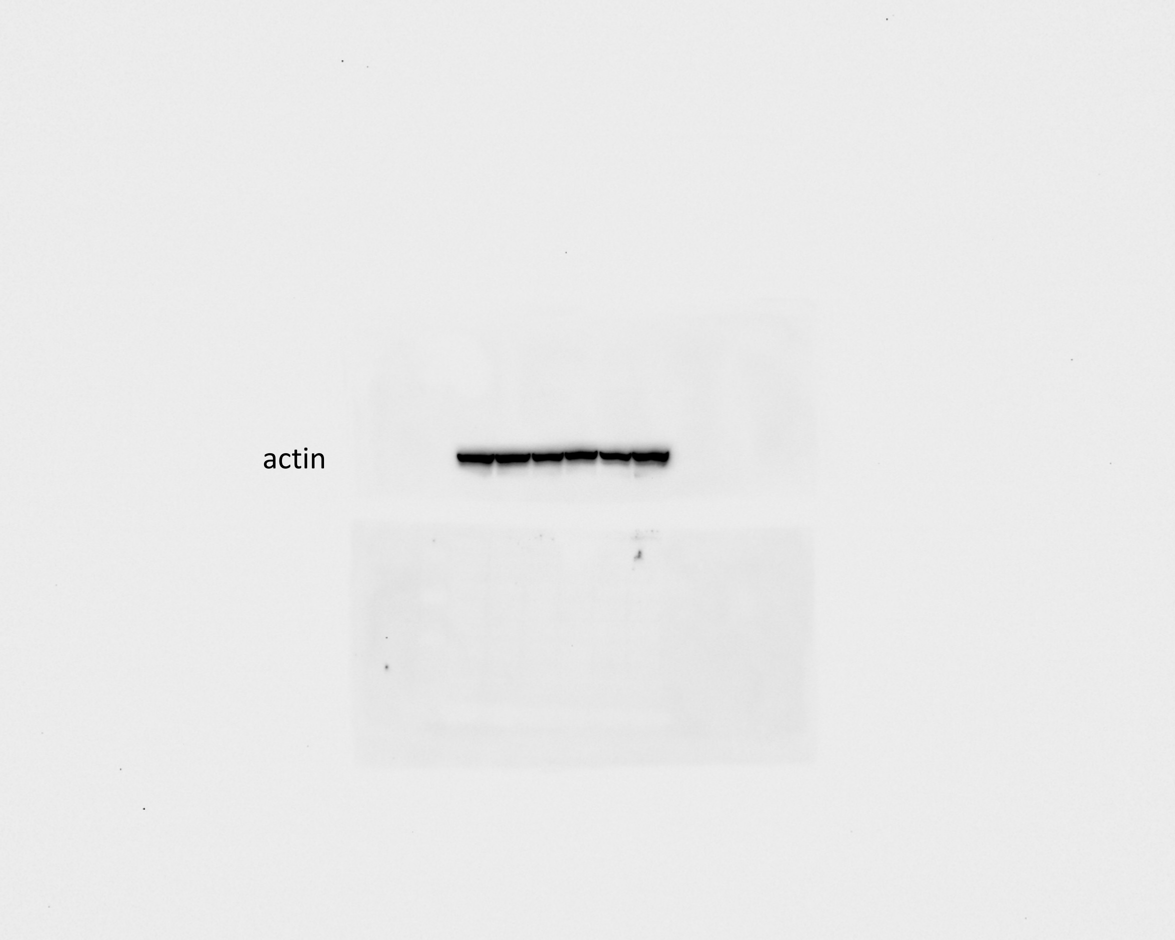

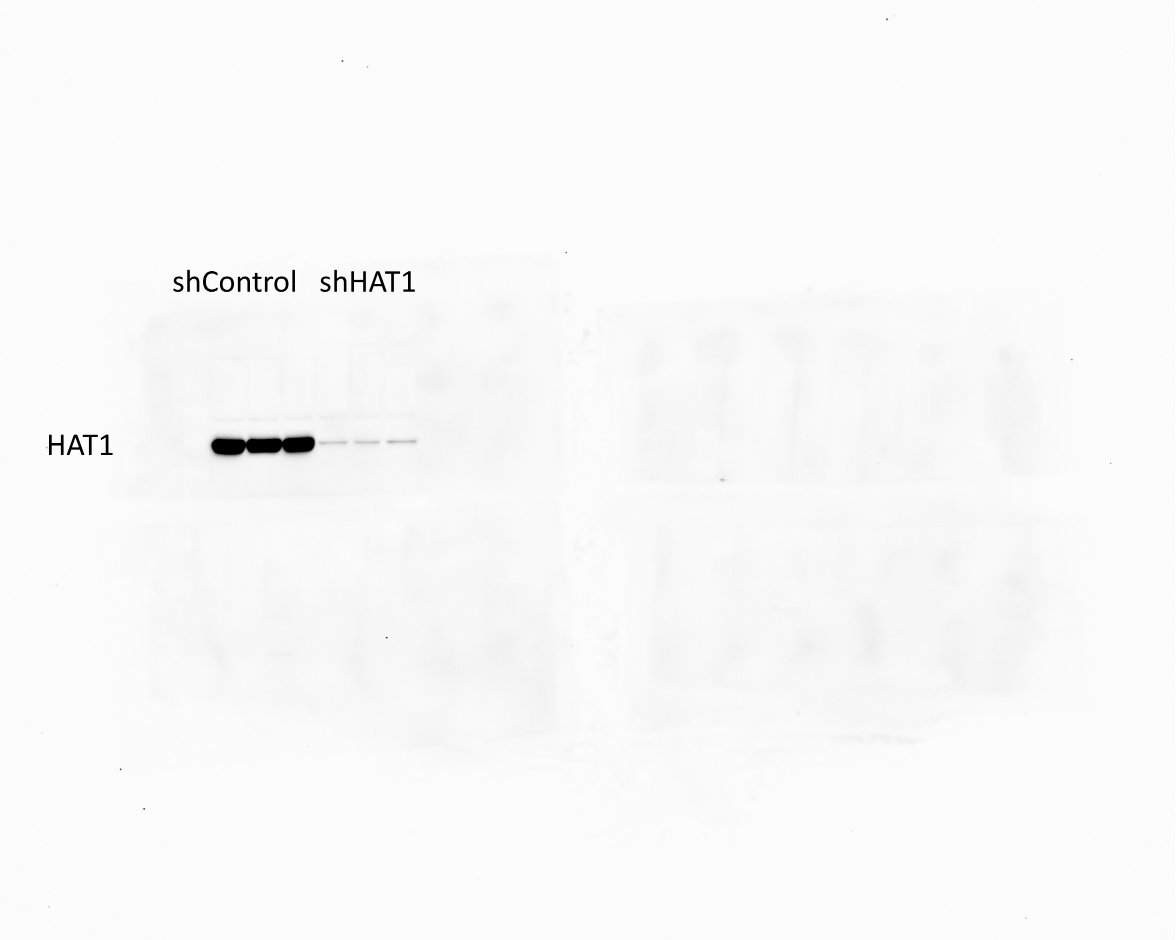

Source Data Extended Data Fig. 9 | Depletion of HAT1 diminishes incorporation of propionate into the H4 lysine 12 site.
